# Thienoisoindigo (TII)‐Based Quinoidal Small Molecules for High‐Performance n‐Type Organic Field Effect Transistors

**DOI:** 10.1002/advs.202002930

**Published:** 2020-11-20

**Authors:** Arulmozhi Velusamy, Chih‐Hsin Yu, Shakil N. Afraj, Chia‐Chi Lin, Wei‐Yu Lo, Chia‐Jung Yeh, Ya‐Wen Wu, Hsin‐Chun Hsieh, Jianhua Chen, Gene‐Hsiang Lee, Shih‐Huang Tung, Cheng‐Liang Liu, Ming‐Chou Chen, Antonio Facchetti

**Affiliations:** ^1^ Department of Chemistry and Research Center of New Generation Light Driven Photovoltaic Modules National Central University Taoyuan 32001 Taiwan; ^2^ Department of Chemical and Materials Engineering National Central University Taoyuan 32001 Taiwan; ^3^ Department of Chemistry and the Materials Research Center Northwestern University Evanston IL 60208 USA; ^4^ Instrumentation Center National Taiwan University Taipei 10617 Taiwan; ^5^ Institute of Polymer Science and Engineering National Taiwan University Taipei 10617 Taiwan; ^6^ Department of Materials Science and Engineering National Taiwan University Taipei 10617 Taiwan

**Keywords:** organic field effect transistors, organic semiconductors, quinoid, solution‐shearing, thienoisoindigo

## Abstract

A novel quinoidal thienoisoindigo (TII)‐containing small molecule family with dicyanomethylene end‐capping units and various alkyl chains is synthesized as n‐type organic small molecules for solution‐processable organic field effect transistors (OFETs). The molecular structure of the 2‐hexyldecyl substituted derivative, **TIIQ‐b16**, is determined via single‐crystal X‐ray diffraction and shows that the **TIIQ** core is planar and exhibits molecular layers stacked in a “face‐to‐face” arrangement with short core intermolecular distances of 3.28 Å. The very planar core structure, shortest intermolecular N···H distance (2.52 Å), existence of an intramolecular non‐bonded contact between sulfur and oxygen atom (S···O) of 2.80 Å, and a very low‐lying LUMO energy level of −4.16 eV suggest that **TIIQ** molecules should be electron transporting semiconductors. The physical, thermal, and electrochemical properties as well as OFET performance and thin film morphologies of these new **TIIQ**s are systematically studied. Thus, air‐processed **TIIQ‐b16** OFETs exhibit an electron mobility up to 2.54 cm^2^ V^−1^ s^−1^ with a current ON/OFF ratio of 10^5^–10^6^, which is the first demonstration of TII‐based small molecules exhibiting unipolar electron transport characteristics and enhanced ambient stability. These results indicate that construction of quinoidal molecule from TII moiety is a successful approach to enhance n‐type charge transport characteristics.

## Graphical abstract

Quinoidal thienoisoindigo (TIIQ)‐based n‐type organic small molecules with various alkyl chains are designed as for solution‐processable organic field effect transistors (OFETs). Among all, **TIIQ‐b16** OFETs yield the highest mobility of 2.54 cm^2^ V^−1^ s^−1^, excellent long‐term ambient stability, and electrical stability.

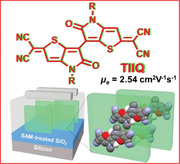

## 1 Introduction

The realization of organic semiconductors (OSCs) for various optoelectronic devices is one of the most active research fields and OSC small molecules have attracted much attention for application in memory devices, smart cards, radio frequency identification tags, transparent circuits, electronic papers, flexible displays, and sensors.^[^ ^1^, ^2^, ^3^, ^4^, ^5^, ^6^, ^7^, ^8^, ^9^, ^10^, ^11^, ^12^, ^13^, ^14^, ^15^ ^]^ OSC unique properties include facile large‐area processing, mechanical flexibility, economically viable production, safer environmental standards, and availability from abundant elements compared to classical and high‐performance inorganic materials. A key performance parameter of an OSC is the charge carrier mobility, which is typically quantified in an organic field effect transistor (OFET) architecture.^[^ ^16^ ^]^ The field‐effect mobility, hereafter defined by *μ*, depends on both intrinsic and extrinsic factors of an OSC, including frontier molecular orbital energetic, molecular structure, and molecular packing characteristics, and OSC film morphological and topological properties.^[^ ^17^, ^18^, ^19^, ^20^, ^21^ ^]^ From a molecular design standpoint, the development of donor–acceptor (D–A)‐type OSCs with conjugated backbone has greatly advanced the OFET technology^[^ ^22^ ^]^ resulting in several high‐performing p‐type (hole‐transporting) and n‐type (electron‐transporting) OSC families^[^ ^7^, ^23^, ^24^, ^25^ ^]^

Despite considerable progress, the performance of n‐type semiconductors continue to lag behind p‐type because of the critical requirements for LUMO energy levels and environmental trapping of electrons.^[^ ^26^, ^27^, ^28^, ^29^, ^30^, ^31^, ^32^ ^]^ Hence, the development of high‐performance and ambient stable n‐type OSCs for OFETs are highly desirable.^[^ ^33^, ^34^, ^35^, ^36^, ^37^ ^]^ It is necessary to design planar conjugated cores/backbones functionalized with suitable side chains, in addition to proper energy levels, to enable high‐performance solution processable n‐type OFETs.^[^ ^38^, ^39^, ^40^, ^41^, ^42^, ^43^ ^]^ The inter and intra molecular interactions, molecular packing, processability, and device performances can be significantly affected by the side chain structure such as, for alkyl groups, chain length, branching position, density on the core, and chirality.^[^ ^44^, ^45^, ^46^, ^47^, ^48^, ^49^, ^50^ ^]^ Aromatic frameworks with conjugated cores functionalized with electron‐withdrawing substituents are suitable building blocks for electron‐transport.^[^ ^51^, ^52^, ^53^ ^]^ Among different OSC structural variations, dicyanomethylene (DCN)‐substituted quinoidal oligothiophenes are excellent n‐type semiconductors because of their high electron affinity originating from the quinoidal structure terminated by two strongly electron withdrawing groups, affording low‐lying LUMO energy levels.^[^ ^54^, ^55^ ^]^ The presence of a *π*‐conjugated quinoidal core is known to strongly modulate the electronic structure.^[^ ^23^, ^56^ ^]^

**Figure 1:**
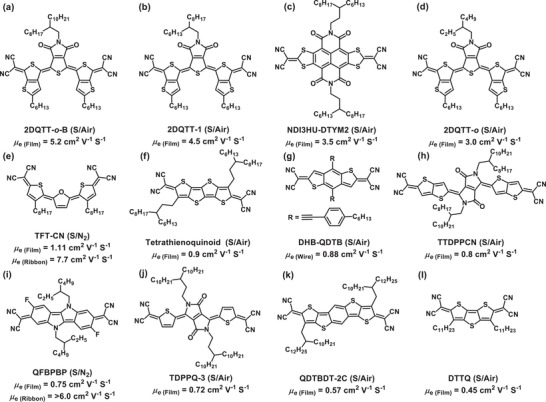
Chemical structures and OFET performances of the reported quinoidal semiconductors incorporating dicyanomethylene end groups. Note, OFETs solution‐processed and measured in ambient condition are denoted as (S/Air) and those solution‐processed and measured under nitrogen are denoted as (S/N_2_).*μ*_e_indicated the maximum electron mobility for the semiconductor in the form of a film, ribbon, or wire.

Fused planar aromatic structures are also known to enhance *π*–*π* stacking and hence induce higher molecular ordering, which may lead to improved device performance with simultaneous increase in charge‐transport properties.^[^ ^57^ ^]^ Thus, several groups have addressed combining (benzo)fusion with the presence of thiophene/S‐moieties in a dicynanomethylene‐substituted quinoidal structure affording organic n‐channel semiconductors with electron mobilities >0.45 cm^2^ V^−1^ s^−1^ (**Figure** **1**). For instance, solution‐processed films of the molecule **2DQTT‐*o*‐B(a)** affords OFETs with the highest electron mobility of 5.2 cm^2^ V^−1^ s^−1[^ ^58^ ^]^ in ambient conditions. In this molecular core, the influence of the alkyl chain have been reported by variation in chain lengths on the nitrogen atom affording electron mobilities up to 4.5 and 3.0 cm^2^ V^−1^ s^−1^ for **2DQTT‐1(b)** ^[^ ^48^ ^]^ and **2DQTT‐*o*(d)**,^[^ ^55^ ^]^ respectively. Another example of core and side‐chain engineering is the NDI‐based quinoidal molecule **NDI3HU‐DTYM2(c)**, which exhibits a maximum electron mobility of 3.5 cm^2^ V^−1^ s^−1^.^[^ ^43^ ^]^ Additional remarkable examples are the n‐type semiconductors **TFT‐CN(e)**,^[^ ^59^ ^]^ **Tetrathienoquinoid(f)**,^[^ ^60^ ^]^ **DHB‐QDTB(g)**,^[^ ^61^ ^]^ **TTDPPCN(h)**,^[^ ^62^ ^]^ **TDPPQ‐3(j)**,^[^ ^42^ ^]^ **QDTBDT‐2C(k)**,^[^ ^41^ ^]^ and **DTTQ(l)**,^[^ ^54^ ^]^ which the corresponding solution‐processed OFETs achieved electron mobilities of 1.11 (under nitrogen), 0.9, 0.88, 0.8, 0.72, 0.57, and 0.45 cm^2^ V^−1^ s^−1^ (in ambient), respectively. Finally, a recent pyrrolo[3,2‐b]pyrrole‐based quinoidal molecule **QFBPBP(i)**, which exhibits an electron mobility of 0.75 cm^2^ V^−1^ s^−1^ (>6 cm^2^ V^−1^ s^−1^ as ribbon) under nitrogen,^[^ ^63^ ^]^ demonstrates the interest of quinoidal pyrrolitic structures.

**Figure 2:**
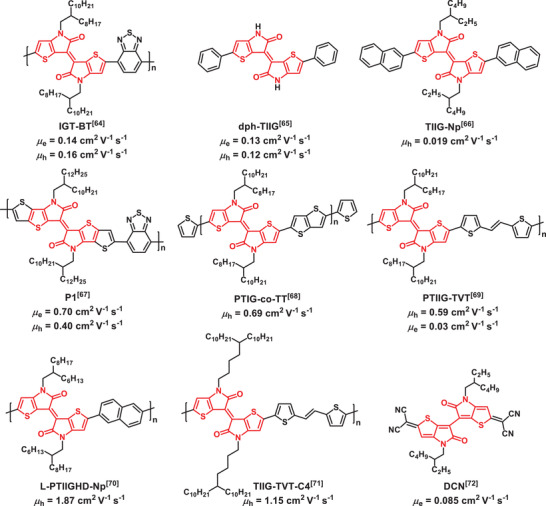
Chemical structures and OFET performances of the thienoisoindigo (TII)‐based small molecules and polymeric OFETs.*μ*_e_and*μ*_h_refer to the maximum electron and hole mobilities, respectively.

**Scheme 1:**
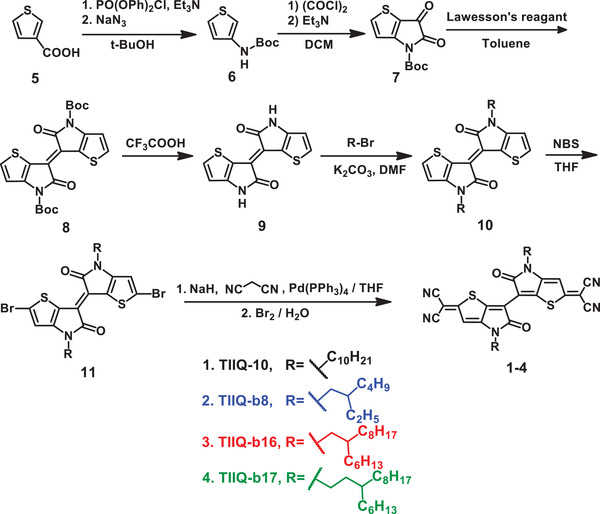
Synthetic route to quinoidal molecules**TIIQ**(**1–4**).

[6,6′‐bithieno[3,2‐*b*]pyrrolylidene]‐5,5′(4*H*,4′*H*)‐dione or thienoisoindigo (TII)‐based structures (**Figure** **2**) have been first investigated by Ashraf et al.,^[^ ^64^ ^]^ and later by Yoo et al.,^[^ ^65^ ^]^ as novel building blocks for both p‐type small molecule^[^ ^66^ ^]^ and polymeric OFETs.^[^ ^67^, ^68^, ^69^, ^70^, ^71^ ^]^ The TII core and the corresponding structures are planar due to thiophene–thiophene links along the backbone, thus maximizing *π*‐conjugation and further enhancing close intermolecular contacts. The strong donor–acceptor character creates a highly hybridized frontier molecular orbital system leading to low‐lying LUMO and high‐lying HOMO orbitals. In an interesting investigation, Chueh et al. implemented TII into a quinoidal structure (**TIIQ‐b8**) affording a maximum electron mobility of 0.085 cm^2^ V^−1^ s^−1^ in ambient conditions.^[^ ^72^ ^]^ Inspired by this seminal work and previous studies showing the importance of alkyl‐chain substitution and film processing to optimize charge transport,^[^ ^54^, ^73^, ^74^ ^]^ here we report several new TII‐based dicyanomethylene *π*‐expanded quinoids (**TIIQ**s (**1–4**); see **Scheme** **1** for molecular structure) for solution‐processable n‐channel OFETs. Further, the optical, electrochemical, and thermal characterizations of all new **TIIQ**s were performed and the results were compared. OSC films were fabricated by solution shearing, considering that it is a reliable technique for enhancing thin film crystallinity and promoting alignment, thus enhancing charge mobility versus isotropic/fast spin‐coating process.^[^ ^54^, ^74^, ^75^ ^]^ Bottom‐gate top‐contact (BGTC) FET device architecture based on solution‐sheared films were fabricated for evaluating the charge transport characteristics. Remarkably, our results revealed that **TIIQ‐b16‐**based OFETs exhibited excellent n‐channel electrical performance, with an electron *μ* as high as 2.54 cm^2^ V^−1^ s^−1^ and a current ON/OFF ratio (*I*_ON_ */I*_OFF_) > 10^5^–10^6^. The crystal structure of one of these molecules, **TIIQ‐b16**, reveal a planar TII core, highly ordered molecular packing, strong *π*–*π* interactions, intramolecular charge transfer between S and O, and intermolecular interaction between N and H, thus enabling large film texturing. The **TIIQ**s film morphologies and microstructures were characterized by optical microscopy (OM), polarized optical microscopy (POM), atomic force microscopy (AFM), and grazing incidence X‐ray diffraction (GIXRD) to correlate them with the device performances.

## 2 Results and Discussion

### 2.1 Synthesis

The synthetic route to quinoid structures (**1–4**) is shown in Scheme 1. The reported synthetic route to thienoisoindigo (TII)^[^ ^64^ ^]^ (Scheme S1, Supporting Information) have been modified to afford good yield with cheaper starting materials as in Scheme 1. 3‐Thiophenecarboxylic acid is treated with *t*‐butanol as a solvent and Curtius rearrangement is carried out with diphenyl phosphoryl chloride and NaN_3_ to obtain Boc‐protected aminothiene **6**. Ring fusing of the latter with oxalyl chloride was carried out in basic condition to give compound (carboxylated thienopyrrole) **7**. Dimerization of **7** with Lawesson's reagent afforded Boc‐protected thienoisoindigo **8**, which was subjected to treatment with trifluoroacetic acid to achieve thienoisoindigo core (**TII**; **9**) in 78% yield. Alkylation of **9** in presence of potassium carbonate gave compound **10**, which was treated with n‐bromosuccinimide to give the corresponding brominated **11**. Via Takahashi coupling of **11** with malononitrile in the presence of tetrakis(triphenylphosphine)palladium, followed by oxidation using a saturated solution of bromine in water, quinoids **TIIQ** (**1–4**) were achieved.

Quinoidal molecules (**1–4**) vary only in side chain type on identical molecular core. Thus, linear and branched alkyl chain of C_10_H_21_ (**a**), bC_8_H_17_ (**b**), bC_16_H_33_ (**c**), and bC_17_H_35_ (**d**) are used in **TIIQ‐10** (**1**), **TIIQ‐b8** (**2**), **TIIQ‐b16** (**3**), and **TIIQ‐b17** (**4**) respectively. Due to the substitution of various alkyl chains, the solubility of the compounds is relatively high in common organic solvents and suitable for thin film solution‐processing.^[^ ^76^, ^77^, ^78^, ^79^ ^]^ All compound chemical structures were characterized by ^1^H NMR, ^13^C NMR, and mass spectrometry. Furthermore, a single crystal X‐ray analysis of **TIIQ–b16** (**3**) was obtained. Synthetic procedure details and characterization data are provided in the Supporting Information.

### 2.2 Physical Characterization

Thermal analyses of the new OSCs were performed using differential scanning calorimetry (DSC; Figure S1a, Supporting Information) and thermogravimetric analysis (TGA; Figure S2, Supporting Information), and the corresponding thermal data and phase transition temperatures are summarized in **Table** **1** and Figure S1b, Supporting Information. From the TGA measurements, it can be seen that all four **TIIQ** molecules exhibit high thermal stability with ≈5% weight loss starting at a temperature in the range of 337–367 °C. As revealed in the DSC scans, all the compounds show clear phase melting transitions. For compound **TIIQ‐b8** functionalized with a short branched alkyl chain, the main melting point is observed at 327 °C and the crystallization peak is seen at 281 °C. Because the side chain on **TIIQ‐b8** is the shortest among the **TIIQ**s, the melting is dominated by the rigid backbone, which explains the high melting temperature. Upon increasing the lengths of the branch chains in **TIIQ‐b16** and **TIIQ‐b17**, the melting temperature decreases since chain motion at higher temperatures reduces core–core intermolecular interactions. Thus, **TIIQ‐b16** shows a sharp melting at 288 °C and a sharp crystallization peak at 254 °C, indicative of the highly ordered crystalline structures. As the branching point of side chains move away from the backbone in **TIIQ‐b17**, the melting point decreases to 218 °C and the peak becomes broader, while the crystallization occurs at 205 °C. Finally, two well‐defined thermal transitions are observed for the analogous linear **TIIQ‐10**, with melting temperatures of 267 and 129 °C and likewise, two crystallization peaks at 233 and 122 °C. This is the result of the presence of linear, and more rigid, alkyl chains on **TIIQ‐10** enabling clear melting of both chain and core, as seen in other molecular systems.^[^ ^80^, ^81^ ^]^ All **TIIQ** phase transition temperatures are given in Figure S1b, Supporting Information.

**Table 1:** Thermal, optical, and electrochemical properties of **TIIQ**s

Compound	*T*_d_ [°C]^a)^	*T*_m_ [°C]^b)^	*λ*_abs_ (soln) [nm]^c)^	*λ*_abs_ (film) [nm]^d)^	△*E*_g_ (opt) [eV]**^e)^**	*E*_ox_ [V]^f)^	HOMO [eV]^g)^, ^h)^	*E*_red_ [V]^f)^	LUMO [eV]^g)^, ^h)^	△*E*_g_ (DPV) [eV]^i)^
**TIIQ‐10**	348	267	587	652	1.77	1.59	−5.79	−0.036	−4.16	1.63
**TIIQ‐b8**	337	327	587	673	1.72	1.60	−5.80	−0.036	−4.16	1.64
**TIIQ‐b16**	367	288	587	666	1.75	1.60	−5.80	−0.036	−4.16	1.64
**TIIQ‐b17**	365	218	587	634	1.80	1.59	−5.79	−0.032	−4.16	1.63

^a)^ Decomposition temperatures were determined from TGA;

^b)^ Melting temperatures were determined from DSC;

^c)^ Absorption spectra were measured in *o*‐C_6_H_4_Cl_2_;

^d)^ Thin films were solution‐sheared onto a quartz glass;

^e)^ The thin film optical energy gap was calculated using 1240/*λ*_abs_ (onset);

^f)^ By DPV in *o*‐C_6_H_4_Cl_2_ at 25 °C. All potentials are reported with reference to an Fc^+^/Fc internal standard (at +0.6 V);

^g)^ Using HOMO/LUMO = −(4.2 + *E*_ox_/*E*_red_);

^h)^ Instead of using HOMO/LUMO, IP/EA may be more appropriate according to ref. [82];

^i)^ The energy gap was calculated from the difference between HOMO and LUMO measured by DPV.

**Figure 3:**
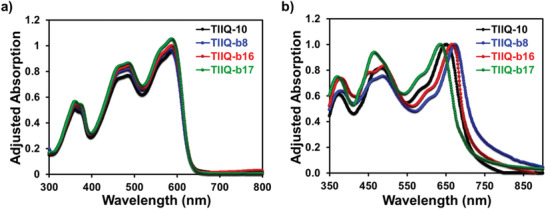
UV–vis absorption spectra of all **TIIQ** compounds in a) solutions and as b) thin films.

The UV–vis absorption spectra both in *o*‐dichlorobenzene and for solution‐sheared films are shown in **Figure** **3**. As seen in Figure 3a, all the solution state absorption spectra are identical and exhibit maximum absorption wavelengths (*λ*_max_) at ≈587 nm, suggesting that variations in side chain substituents do not have strong influences on the *π*‐conjugated thienoisoindigo (**TII**) backbone. The absorption maximum wavelengths and optical bandgaps determined from the film absorption are summarized in Table 1. The absorption peak shapes of the four materials are almost identical. Clearer vibronic absorption features proved a proof for an increased aggregation for **TIIQ‐b8** and **TIIQ‐b16** branched small molecules than **TIIQ‐b17** and **TIIQ‐10**. In addition, the optical bandgaps of these compounds were determined from the onset of the absorption spectrum of the films, and found to be 1.77, 1.72, 1.75, and 1.80 eV for **TIIQ‐10**, **TIIQ‐b8**, **TIIQ‐b16,** and **TIIQ‐b17**, respectively. The red‐shift absorption with a lower optical bandgap in **TIIQ‐b8** and **TIIQ‐b16** might be due to a combination of different extent of aggregation and different electronic coupling and packing modes.

**Figure 4:**
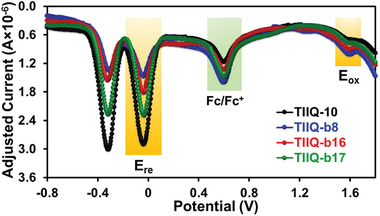
Oxidation potential curves of all**TIIQ**s in*o*‐dichlorobenzene. All potentials reported are referenced to an Fc/Fc^+^internal standard (at + 0.6 V).

Molecular energy levels of four **TIIQ**s examined by differential pulse voltammetry (DPV) measurements are shown in **Figure** **4** and Table 1. The DPV data show that all the quinoidal molecules possess similar oxidation and reduction potentials, and thus alkyl substituents of the conjugated backbones have no significant effect on the electrochemical properties of the **TIIQ**s. Consequently, the derived lowest unoccupied molecular orbital (LUMO) and the highest occupied molecular orbital (HOMO) of **1**–**4** are located at around −4.16 and −5.80 eV, respectively, according to the equation: HOMO/LUMO = −(4.2 + *E*_ox_ */E*_red_); assuming an internal standard ferrocene/ferrocenium (Fc/Fc^+^) oxidation at −4.8 eV. The low‐lying LUMO energy level of these **TIIQ**s should lead to facile electron injection in OFETs and prove the potential ambient stability for these n‐type dicyanomethylenyl **TII** quinoids. The electrochemically derived HOMO‐LUMO energy gaps of the **TIIQ**s are ≈1.64 eV, which is very close to those determined by UV–vis measurements.

### 2.3 Theoretical Calculations

The backbone geometries and frontier molecular orbitals of **TIIQ** compounds were calculated at the B3LYP/6‐31G* level of density functional theory (DFT) using Gaussian 09 program. As illustrated in Figure S3, Supporting Information, all four compounds have similar FMO surface topologies where the electron densities are delocalized on the whole conjugated units, which display good electronic overlapping. From the DFT calculations, all the HOMO/LUMO energy levels are calculated to be −4.12 to −4.16 eV/−6.34 to −6.38 eV. These results are consistent with the DPV measurements.

### 2.4 Single Crystal Structure

**Figure 5:**
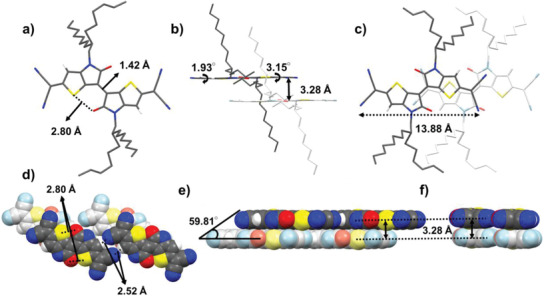
Single crystal structure of**TIIQ‐b16**(**3**) in stick models (a–c) and space filling packing models (d–f). a) Top view of**TIIQ‐b16**with intramolecular S···O distance of 2.80 Å and the C‐C distance between two TP molecules in TII is 1.42 Å. b) Front view of**TIIQ‐b16**in a stick model with the torsion angle of 1.93 Å to 3.15 Å. c) Top view of**TIIQ‐b16**and the molecular length of 13.88 Å. d) Shortest intermolecular N···H distance of 2.52 Å. e,f) Molecular packing arrangement of**TIIQ**with face‐to‐face layer stacking distance of 3.28 Å and exhibits the slipping angle of 59.81°.

Single crystals of **TIIQ‐b16** were obtained from a chlorobenzene–methanol solvent mixture by slow solvent evaporation. The diffraction‐derived single‐crystal structure of **TIIQ‐b16**, as the representative of this family, is shown in **Figure** **5** and Figure S4, Supporting Information, and detailed crystal structure data are summarized in Table S1, Supporting Information. As shown in Figure 5, **TIIQ‐b16** recrystallizes in the triclinic space group of P‐1. Shorter S···O distance of 2.80 Å showed the existence of an intramolecular non‐bonded interaction between sulfur and oxygen atoms (Figure 5a). Both TP groups are connected by the C‐C bond with the distance of 1.42 Å, exhibiting the characteristic of a double bond (Figure 5a). The **TIIQ** main core and the two end‐capped CN groups are nearly coplanar, showing small torsional angles of 1.93–3.15 ° (Figure 5b). The molecular length of **TIIQ‐b16** is 13.88 Å. (Figure 5c). Shortest intermolecular N···H distance of 2.52 Å between two **TIIQ** layers increases the order of *π*–*π* molecular stacking (Figure 5d) and measured intermolecular distance of **TIIQ‐b16** between N···H shown in Figure S5, Supporting Information. The **TIIQ** molecules possess brick‐type stack molecular packing arrangement (Figure 5d). Viewed along the short axis *π*‐plane direction, **TIIQ** layer exhibits a slipping angle of 59.81° (Figure 5e). The short intermolecular stacking distance of closely packed TII core is 3.28 Å (Figure 5e,f). In summary, the planar molecular structure, short main‐core stacking distance (3.28 Å), and short intermolecular N···H distance (2.52 Å) of **TIIQ‐b16** suggest ideal conditions for the extended *π*‐orbital interaction of the corresponding molecule, resulting in highest device performance (vide infra).

### 2.5 Charge Transport Properties

**Figure 6:**
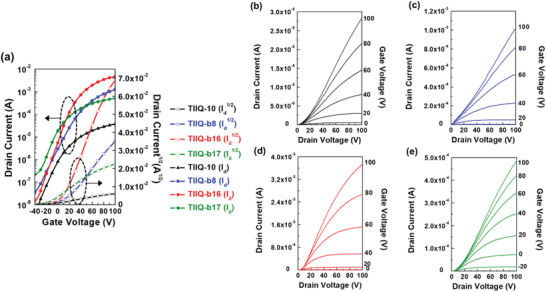
a) Representative transfer characteristics and b–e) output characteristics of**TIIQ‐10**,**TIIQ‐b8**,**TIIQ‐b16**, and**TIIQ‐b17**, respectively, for charge transport parallel to the shearing direction.

To investigate the impact of the side chain on the charge transport properties of the **TIIQ** compounds, BGTC OFETs were fabricated and characterized. The OSC solutions were solution‐sheared onto the PETS‐modified Si/SiO_2_ substrates and the resulting films thermally annealed at 80 °C under vacuum. The details regarding OFET fabrication and characterization are reported in Experimental Section. The OFET mobilities were first evaluated in the parallel direction (//) with respect to the semiconductor shearing direction, which in previous studies was found to correspond to efficient charge transport along the axial direction of the aligned crystals.^[^ ^83^, ^84^, ^85^, ^86^ ^]^ Representative transfer and output characteristics of **TIIQ**‐based OFETs are shown in **Figure** **6**a and 6b–e, respectively. Device parameters such as the maximum/average field effect mobility (*μ*_max_/*μ*_avg_), ON/OFF current ratio (*I*_ON_/*I*_OFF_), and threshold voltage (*V*_th_) are listed in **Table** **2**. All the transfer curves in Figure 6a were measured by sweeping *V*_g_ −40 to 100 V with a fixed *V*_d_ of 100 V. A positive *V*_g_ modulates the ON‐current, indicating a typical n‐channel OFET response. The output curves (Figure 6b–e) exhibit a characteristic transition to saturation behavior and pitch‐off with increasing *V*_g_. The electron mobility was calculated using the gradient from a plot of the square root of *I*_d_ against *V*_g_ in the transfer curve. Among all semiconductors, the electron mobility of **TIIQ‐b16** was found to be the highest. The *μ*_max_ and *μ*_avg_ of **TIIQ‐b16** can reach 2.54 and 1.14 ± 0.45 cm^2^ V^−1^ s^−1^, respectively, which is ≈3×, 10×, and 100× higher than those of **TIIQ‐b8**, **TIIQ‐b17,** and **TIIQ‐10**, respectively. The **TIIQ‐b16** OFETs exhibit excellent *I*_ON_/*I*_OFF_ of 10^5^–10^6^. Clearly, the semiconducting performance can be dramatically enhanced by introducing a branch side chain with increasing length and a branch point close to the conjugated backbone.

**Table 2:** Summary of OFET parameters based on solution‐sheared **TIIQ**s thin films

	Thin film (**//**)^a)^				Thin film (⊥)^a)^			
Compound	*μ*_max_ ^b)^ [cm^2^ V^−1^ s^−1^]	*μ*_avg_ ^c)^ [cm^2^ V^−1^ s^−1^]	*I*_ON_ */I*_OFF_ [–]	*V*_th_ ^b)^ [V]	*μ*_max_ ^b)^ [cm^2^ V^−1^ s^−1^]	*μ*_avg_ ^c)^ [cm^2^ V^−1^ s^−1^]	*I*_ON_ */I*_OFF_ [–]	*V*_th_ ^b)^ [V]
**TIIQ‐10**	0.013	0.006 ± 0.003	10^3^–10^4^	−6.61 ± 3.89	0.009	0.005 ± 0.002	10^3^–10^4^	−0.14 ± 6.34
**TIIQ‐b8**	0.792	0.462 ± 0.165	10^4^–10^5^	−1.26 ± 2.99	0.462	0.228 ± 0.107	10^4^–10^5^	−4.39 ± 3.96
**TIIQ‐b16**	2.54	1.14 ± 0.454	10^5^–10^6^	16.2 ± 7.23	0.27	0.15 ± 0.066	10^5^–10^6^	18.2 ± 3.90
**TIIQ‐b17**	0.195	0.113 ± 0.035	10^3^–10^4^	−27.0 ± 6.64	0.18	0.118 ± 0.029	10^3^–10^4^	−33.6 ± 3.26

^a)^ Charge transport direction is symbolized by // (parallel) and ⊥ (perpendicular) with respect to the solution‐shearing direction;

^b)^ Maximum mobility;

^c)^ Average mobility.

**TIIQ**‐based OFETs were also characterized for charge transport perpendicular (⊥) to the shearing direction to extract the corresponding perpendicular mobility. According to the output and transfer curves of **TIIQ** OFETs in the perpendicular direction, no significant changes in ON‐current and mobility values were recorded for the **TIIQ‐b17** and **TIIQ‐10** devices, while those of the **TIIQ‐b8** FETs decrease by ≈ 2× between these two directions. On the other hand, a *μ*_max_ of 0.27 cm^2^ V^−1^ s^−1^ was attained along the perpendicular direction in **TIIQ‐b16** OFETs, which is almost 10× lower than those measured in the parallel direction. As summarized in Table 2, for this device, the perpendicular *μ*_avg_ is 0.15 ± 0.07 cm^2^ V^−1^ s^−1^ with and *I*_ON_/*I*_OFF_ of 10^5^–10^6^. The observed charge transport anisotropy (the ratio between parallel and perpendicular mobility) is thus determined to be ≈8. The trend of highly efficient and anisotropic transport in the **TIIQ‐b16** film is consistent with the results of morphological and microstructural studies, which will be discussed in the following section.

Finally, note that the *I*–*V* characteristics clearly show that the overall performance trends reflect the mobility trend. Obviously, the devices based on our solution‐sheared films have considerable contact resistance, clearly shown in the output plots of Figure 6, which prevent comparison of the sub‐threshold behavior. However, the carrier mobility in saturation is less affected and tracks current variations in all devices. Furthermore, we have also analyzed the mobility evolution of a representative device of **TIIQ‐b16**, as shown in the Figure S6, Supporting Information. The mobility‐*V*_g_ plot is also very common and approaches the saturation mobility number after turn‐on, typical of a close‐to‐ideal FET.^[^ ^87^ ^]^

### 2.6 Thin Film Morphology and Structural Analysis

**Figure 7:**
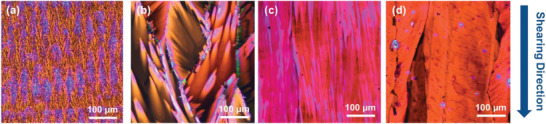
POM image of solution‐sheared a)**TIIQ‐10**, b)**TIIQ‐b8**, c)**TIIQ‐b16**, and d)**TIIQ‐b17**.

POM images of the four **TIIQ** films fabricated through the solution‐shearing method are shown in **Figure** **7**. In the case of **TIIQ‐10** with linear alkyl side chains, instead of mid/large crystalline domains, a very thin film with discontinuous fibrotic features form along the shearing direction (Figure 7a), a morphology unsuitable for a conducting channel. The **TIIQ‐b8** film exhibits a ribbon‐like morphology without preferential macroscopic crystal growth direction (Figure 7b). The lower solubility caused by the shorter side chain in **TIIQ‐b8** leads to early seeding during the shearing process resulting in a randomly oriented and irregular film morphology, which could trap charges. In contrast, a continuous film with textured features along the shear direction can be observed for **TIIQ‐b16**, revealing a highly oriented and elongated crystalline structure (Figure 7c) that facilitates the transportation of charge carriers. **TIIQ‐b17** shows formation of large crystalline sheets roughly oriented along the shearing direction, on which small cracks form in the perpendicular direction that could greatly interrupt the movement of charge carrier (Figure 7d). Since we prepared films with similar concentrations/solvent, it is certain that upon shear deposition, **TIIQ‐b8** and **TIIQ‐10** crystallize much faster than those of the other two molecules, resulting in reduced texturing.

**Figure 8:**
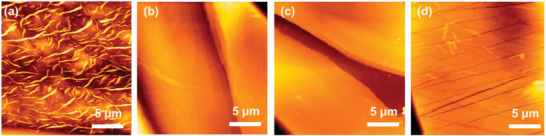
AFM image of solution‐sheared a)**TIIQ‐10**, b)**TIIQ‐b8**, c)**TIIQ‐b16**, and d)**TIIQ‐b17**.

A closer inspection of the **TIIQ** films was probed by AFM as shown in the height images of **Figure** **8**. The strong *π*–*π* interactions between the neighboring **TIIQ** cores and van der Walls interaction between the alky chains enable **TIIQ‐b8** and **TIIQ‐b16** molecules to self‐organize into large and interconnected sheets with a smooth surface. Interestingly, the cracks observed on the **TIIQ‐b17** sheets and the discontinuous fibrils in **TIIQ‐10** film can be more clearly seen in the AFM images, indicating a lack of the continuity of the crystalline domains, which fully agrees with the corresponding low electron mobilities.

Finally, note that **TIIQ‐b16** devices were also fabricated by spin‐coating the semiconductor and the transfer/output curves are shown in Figure S7,Supporting Information. These devices exhibit a mobility which is ≈25‐fold lower than those measured by the shearing process. This is the result of formation of far smaller crystallines as shown in in Figure S8, Supporting Information. Thus, we believed that enhanced charge transport by shearing in this family, as seen previously for other semiconductor classes, is mainly due to enhanced size and alignment of continuous crystal domains, as we will see in mode details later.

**Figure 9:**
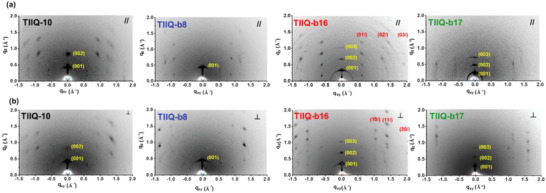
2D GIXRD diffraction patterns of solution‐sheared**TIIQ**s thin films. The incident beam was controlled to be a) parallel and b) perpendicular to the shearing direction.

GIXRD measurements were carried out to investigate how the side chain substituents affect the crystallinity and molecular orientation of the **TIIQ** films. **Figure** **9** shows the 2D GIXRD patterns probed with the incident beam parallel and perpendicular to the shearing direction. The (00*l*) lamellar diffractions in the out‐of‐plane (*q*_z_) direction are clearly detected for all the films, especially for **TIIQ‐b16** and **TIIQ‐b17**, that show distinct (003) peaks, implying a dominated edge‐on molecular orientation. The side chains are aligned normal to the substrate and the **TIIQ** conjugated backbones are aligned parallel to the substrate, which is caused by the strong **TIIQ** intermolecular interaction and is beneficial for lateral charge transport in the OFET channel. **TIIQ‐b8** only shows the (001) diffraction peak without higher order ones, indicating a less ordered layered structure and a lower crystallinity, while **TIIQ‐10** exhibits moderate crystal orientation with a (001) diffraction pattern located both in the out‐of‐plane and in‐plane directions. The lamellar spacing of **TIIQ‐10**, **TIIQ‐b8**, **TIIQ‐b16,** and **TIIQ‐b17**, and calculated from the primary peak are 14.7, 13.5, 18.5, and 22.3 Å, respectively, which are consistent with the lengths of the side chains.

More structural information can be obtained from the 1D profiles along the out‐of‐plane direction extracted from the GIXRD patterns (Figure S10, Supporting Information). **TIIQ‐b16** and **TIIQ‐b17** exhibit sharper diffraction peaks of the lamellar stacking, which generally reflects a larger crystal grain size. The crystal coherence length (*L*_c_), an indication of the grain size, can be determined by the full width at half maximum (FWHM) of the diffraction peaks through Scherrer equation as 2*π*/FWHM.^[^ ^88^ ^]^ The microstructural *L*_c_ can be used for comparison of crystallite domain size present in the samples. The FWHM values are 0.042, 0.035, 0.022, and 0.016 Å^−1^ for **TIIQ‐10**, **TIIQ‐b8**, **TIIQ‐b16**, and **TIIQ‐b17** films, respectively, yielding *L*_c_ of 134.0, 161.5, 252.2, and 349.5 Å. A larger grain size indicates less grain boundary that hinders the charge transport. Therefore, the larger *L*_c_ of **TIIQ‐b16**, along with the strong diffraction that reveals a high crystallinity and a highly ordered crystal structure, explains the high electron mobility of **TIIQ‐b16** in OFET. Although the *L*_c_ of **TIIQ‐b17** is the largest, the lower mobility is in part due to the lower crystallinity and, more likely, cracks forming in the crystals as clearly imaged by both AFM and POM.

To further understand the packing orientation in the **TIIQ** films, the (001) diffraction intensity as a function of azimuthal angle, that is, the pole figure, is examined, as shown in Figure S11, Supporting Information. The integrated area with azimuthal angle from –45**°** to 45**°** is accounted the portion of the edge‐on orientation while the rest corresponds to the face‐on orientation. The fractions of the edge‐on orientation calculated from the pole figures are in the following order: **TIIQ‐b16** (94.47%) > **TIIQ‐b17** (87.40%) > **TIIQ‐b8** (75.16%) > **TIIQ‐10** (69.73%). The edge‐on orientation is normally suitable for the charge transport in OFETs due to the lateral charge delivery in the *π*‐stacking channel between the source and drain contact. Therefore, the high fraction of edge‐on orientation in **TIIQ‐b16** films also further contributes to the measured high electron mobility.

The comparison of the 2D GIXRD patterns with the incident X‐ray beam parallel and perpendicular to the shearing direction in Figure 9 reveals a pronounced difference for the **TIIQ‐b16** film in these two directions while quite similar diffraction patterns are seen for the other **TIIQ** films. The **TIIQ‐b16** film shows the (01*l*), (02*l*), and (03*l*) diffractions, that is, the periodic structure along the conjugated backbone in the *b*‐axis, for the parallel direction, whereas these diffractions are absent for the perpendicular direction and, instead, the (10*l*), (11*l*), (20*l*) diffraction peaks which correspond to the side stacking of the conjugated backbones appear only in the perpendicular direction. These results demonstrate that the **TIIQ‐b16** film is highly anisotropic after shearing, with the *π*‐stacking direction approximately oriented parallel to shearing direction, while other films are rather isotropic even under shearing. The aligned *π*‐stacking provides a low‐barrier pathway for charge carriers to transport. This explains why the **TIIQ‐b16** film shows an electron mobility along the shearing direction one order of magnitude higher than that perpendicular to the shearing direction. Although there is only one carbon atom variation between the side chains in **TIIQ‐b16** and **TIIQ‐b17**, the film morphology, crystallinity, and orientation are significantly different, manifesting the impact of the side chains on film morphology and charge transport.

In conclusion, since our deposition methodology align the crystal in the *π*‐stacking direction, it is conceivable to conclude that enhanced transport and anisotropy are the result of alignment. Also, it is known that if no cracks form in a thin‐film of an OSC, then enhanced crystallinity/crystal dimension improves overall charge transport. Thus, the overall transport observation correlates quite well and is consistent with our microstructural and morphological analysis. Further theoretical calculation can be considered such as transfer integral, electronic coupling, and effective mass among the adjacent molecules from single crystal state of OSCs to support the anisotropic charge transport properties.^[^ ^89^, ^90^, ^91^ ^]^

### 2.7 OFET Stability

**Figure 10:**
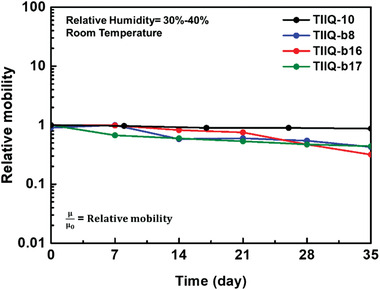
Ambient stability of**TIIQ**s OFETs stored at relative humidity of 30–40% and room temperature.

Realistic OFET applications require stable charge transport, which is evidenced by stable *I–V* response upon operational and environmental stresses. In order to characterize the reliability of the **TIIQ** OFETs, the electrical sweeps in the transfer curves for *V*_g_ from −40 to 100 V were repeatedly scanned (Figure S12, Supporting Information). With increasing the operating cycles, the *V*_th_ slightly shifts to the positive voltage direction with negligible ON‐current change. Therefore, charge trapping at the interface between the OSCs and passivated gate dielectric as well as dipole polarization can be excluded in these OFETs.^[^ ^92^, ^93^, ^94^ ^]^ Tailoring the chemical structures of an OSC is an effective strategy to improve the intrinsic environmental stability. It has been theoretically proven and empirically demonstrated that a LUMO energy level of –4.0 eV or lower is necessary to realize stable electron transport in n‐type OSCs for FETs.^[^ ^95^, ^96^ ^]^ Thanks to the strong electron‐withdrawing TII core and cyano end‐substituents, the LUMO energy of these compounds is about −4.16 eV. When the **TIIQ** OFETs are exposed to the ambient air (relative humidity of 30–40%; at room temperature), the devices maintain decent semiconducting performance for 35 days (**Figure** **10**). Although **TIIQ‐10** exhibits more stable field‐effect mobility than the other three semiconductors, it is also important to stress that this semiconductor also affords the lowest FET mobility, far lower than those of the other devices. Thus, less current is injected into the channel, which can reduce possibility of device (particularly contact) degradation.^[^ ^92^, ^93^, ^94^ ^]^ Furthermore, this semiconductor has a very different film morphology than the others. Regarding the other three, statistically, the FET mobility variations are practically identical. Thus, combining strong electron‐withdrawing building blocks and suitable side chain substituents can achieve semiconducting molecules with good solution processability and efficient electron‐transport in OFETs. Simple film passivation can improve stability even further.

## 3 Conclusions

In conclusion, we demonstrated new quinoidal semiconducting molecules, **TIIQ**s (**1–4**), some of them combining proper electronic structures, solubility parameters, and well‐connected crystal domains for high‐performance n‐type OFET applications. Charge transport in these molecules was rationalized by combining morphological and microstructural data. Particularly, **TIIQ‐b16** based OFETs exhibited excellent n‐channel electrical performance, with a *μ* as high as 2.54 cm^2^ V^−1^ s^−1^ and an *I*_ON_ */I*_OFF_ > 10^5^–10^6^ as well as quite stable transport characteristics. This is due to the formation of large and well‐connected crystallites along the shear direction, which also explains the measured charge transport anisotropy. The crystal structure of **TIIQ‐b16** also corroborates the favorable electron transport due to the presence of a planar TII core, highly ordered and close *π*–*π* packing, and close contact between the heteroatom favoring planarization and close molecular proximity. Overall, this study demonstrates that these quinoidal motifs, despite the extremely small *π*‐conjugated core, can offer a path to achieve solution‐processable n‐type OSC‐based devices.

## 4 Experimental Section

##### Materials

All the chemical reagents were purchased from Aldrich, Alfa, and TCI Chemical Co. and used as received unless otherwise noted. Solvents for reactions (toluene and THF) were distilled under nitrogen from sodium/benzophenone ketyl, and halogenated solvents were distilled from CaH_2_. Silane agent for the self‐assembled monolayer (SAM) treatment (2‐phenylethyl)trichlorosilane (PETS) or octadecyltrimethoxysilane (ODTS) was obtained from Gelest, Inc. Details of the preparation of intermediates from **6** to **11** are shown in Supporting Information.

##### General Procedures for Final Target Compounds (**1**–**4**)

Malononitrile (1.15 mmol) was added into a solution of sodium hydride (2.29 mmol) in dry THF (20 mL) at 0 °C, and the mixture was warmed to room temperature and stirred for 30 min. (*E*)‐2,2′‐dibromo‐4,4′‐dialkyl‐[6,6′‐bithieno[3,2‐*b*]pyrrolylidene]‐5,5′(4*H*,4′*H*)‐dione (**9**) (0.28 mmol) and tetrakis(triphenylsphosphine)palladium (0.06 mmol) were then added. The mixture was refluxed for 12 h, and then saturated bromine water was added at 0 °C and stirred for 20 min. The mixture was extracted with CH_2_Cl_2_, washed with brine, dried over Na_2_SO_4_, and evaporated. The residue was purified by column chromatography using dichloromethane/hexane followed by recrystallization from n‐hexane.

##### Synthesis of **TIIQ‐10** (**1**)

The title compound was obtained as a dark red solid (yield = 65%). Mp: 267 °C. ^1^H NMR (500 MHz, CDCl_3_): *δ* 6.53 (s, 2 H), 3.72 (t, *J* = 7.5 Hz, 4 H), 1.67 (m, 4 H), 1.4–1.2 (m, 28 H), 0.87 (t, *J* = 7 Hz, 6 H). ^13^C NMR: This compound was insufficiently soluble to obtain a useful ^13^C NMR spectrum. HRMS (MALDI, [M+H]^+^) calcd. for C_38_H_45_N_6_O_2_S_2_: 681.3040. Found: 681.3040.

##### Synthesis of **TIIQ‐b8** (**2**)

The title compound was obtained as a dark red solid (yield = 64%). Mp: 327 °C. ^1^H NMR (500 MHz, CDCl_3_): *δ* 6.48 (s, 2 H), 3.61 (d, *J* = 7.5 Hz, 4 H), 1.77 (s, 2 H), 1.32 (m, 16 H), 0.93 (t, *J* = 7.5 Hz, 12 H). ^13^C NMR (125 MHz, CDCl_3_): *δ*174.77, 170.59, 159.05, 143.65, 114.19, 112.24, 112.07, 104.01, 75.12, 46.37, 38.34, 30.29, 28.35, 23.74, 22.99, 14.00, and 10.39. HRMS (FAB, [M+H]^+^) calcd. for C_34_H_37_N_6_O_2_S_2_: 625.2414. Found: 625.2410.

##### Synthesis of **TIIQ‐b16** (**3**)

The title compound was obtained as a dark red solid (yield = 52%). Mp: 288 °C. ^1^H NMR (500 MHz, CDCl_3_): *δ* 6.47 (s, 2 H), 3.60 (d, *J* = 7.5 Hz, 4 H), 1.82 (s, 2 H), 1.26 (m, 48 H), 0.87 (t, *J* = 3.5 Hz, 12 H). ^13^C NMR (125 MHz, CDCl_3_): *δ* 174.72, 170.59, 159.06, 143.65, 114.17, 112.22, 112.06, 104.00, 75.15, 46.72, 37.11, 31.86, 31.73, 31.23, 29.93, 29.59, 29.48, 29.26, 26.19, 26.11, 22.66, 22.61, and 14.11. HRMS (MALDI, [M+H]^+^) calcd. for C_50_H_69_N_6_O_2_S_2_: 849.4918. Found: 849.4918.

##### Synthesis of **TIIQ‐b17** (**4**)

The title compound was obtained as a dark red solid (yield = 52%). Mp: 218 °C. ^1^H NMR (500 MHz, CDCl_3_): *δ* 6.50 (s, 2 H), 3.72 (t, *J* = 7.5 Hz, 4 H), 1.60 (m, 6 H), 1.26 (m, 48 H), 0.87 (m, 12 H). ^13^C NMR (125 MHz, CDCl_3_): *δ* 174.72, 170.21, 158.51, 143.64, 114.24, 112.22, 112.03, 103.78, 75.18, 40.61, 35.38, 33.28, 32.18, 31.88, 31.85, 29.96, 29.61, 29.32, 26.56, 26.50, 22.67 and 14.10. HRMS (MALDI, [M+H]^+^) calcd. for C_52_H_73_N_6_O_2_S_2_: 877.5231. Found: 877.5183.

##### Characterization

All the target compounds were analyzed by ^1^H NMR and ^13^C NMR spectroscopy recorded on Bruker 500 or 300 instrument using CDCl_3_ as the solvent. Elemental analyses were performed on a Heraeus CHN‐O‐Rapid elemental analyzer. Mass spectrometric data were obtained with a JMS‐700 and ATS‐00670 HRMS instrument. DSC was carried out under a nitrogen atmosphere on a Mettler DSC 822 instrument (scanning rate of 10 °C min^−1^). TGA was carried out using a Perkin Elmer TGA‐7 thermal analysis system using dry nitrogen as a carrier gas at a flow rate of 10 mL min^−1^ (heating rate of 10 °C min^−1^), and the reported decomposition temperatures represent the temperature observed at 5% mass loss. DPV experiments were performed with a conventional three‐electrode configuration (a platinum disk working electrode, an auxiliary platinum wire electrode, and a non‐aqueous Ag reference electrode), with a supporting electrolyte of 0.1 m tetrabutylammonium hexafluorophosphate (TBAPF_6_) in the specified dry solvent using a CHI621C Electrochemical Analyzer (CH Instruments). All electrochemical potentials were referenced to an Fc/Fc^+^ internal standard (at 0.6 V). The UV–vis spectrum was characterized with a JASCO V‐670 UV–vis spectrophotometer. Polarized optical images were measured with a Leica 2700M. The tapping mode AFM images were recorded using Seiko SPA‐400 and acquired using a Si cantilever with resonance frequency of 260 kHz and spring constant of 30 N m^−1^. Synchrotron‐based GIXRD was performed at the B13A, 17A1, and 23A1 beamlines at National Synchrotron Radiation Research Center (NSRRC, Taiwan) to investigate the crystallinity and molecular orientation.

##### Device Fabrication and Measurement

Highly n‐doped Si wafers with 300‐nm‐thick thermally grown SiO_2_ were sonicated for 5 min each time by acetone and isopropanol, dried with nitrogen, and treated with UV ozone plasma for 5 min. They were used as substrate/gate electrode/gate dielectric and immersed into PETS solution in toluene (1 µL mL^−1^) at 55 °C for 1 h to form the SAM. A programmable custom‐built shearing apparatus was used for the deposition of the OSC crystalline film. The substrate was fixed at the bottom plate by vacuum whereas the top blade could be moved relative to the substrate by servomotor. The OSC films could be deposited on the heated platform (90–120 °C) at a controlled shearing speed (15–170 µm s^−1^). The samples were then thermally annealed at 80 °C for 1 h under vacuum. After laminating the shadow mask, silver source and drain electrode were deposited at 0.5 Å s^−1^ under a pressure of below 4 × 10^−6^ torr in a thermal evaporation chamber. The electrodes were designed with patterns oriented along both the parallel and perpendicular directions versus the shearing direction. The channels were 25 µm in length and 1500 µm in width. The electrical characteristics of the OFETs were measured by Keithley 4200‐SCS combined probe station at room temperature inside a N_2_‐purged glove box in the dark or in ambient.

## Conflict of Interest

The authors declare no conflict of interest.

## Supporting information

Supporting Information
