# Supplementary material for: Thienoisoindigo (TII)‐Based Quinoidal Small Molecules for High‐Performance n‐Type Organic Field Effect Transistors
Source: Adv Sci (Weinh). 2020 Nov 20;8(1):2002930. doi: 10.1002/advs.202002930 (PMC7788596; doi:10.1002/advs.202002930)
Supplement: Supplementary file 1 — Supporting Information [file ADVS-8-2002930-s001.pdf]

© 2020 Wiley-VCH GmbH

ADVANCED SCIENCE
Open Access

Supporting Information

for *Adv. Sci.,* DOI: 10.1002/advs.202002930

Thienoisoindigo (TII)-Based Quinoidal Small Molecules for High-Performance n-Type Organic Field Effect Transistors

Arulmozhi Velusamy, Chih-Hsin Yu, Shakil N. Afraj, Chia-Chi Lin, Wei-Yu Lo, Chia-Jung Yeh, Ya-Wen Wu, Hsin-Chun Hsieh, Jianhua Chen, Gene-Hsiang Lee, Shih-Huang Tung, Cheng-Liang Liu, Ming-Chou Chen,* and Antonio Facchetti**

© Copyright WILEY-VCH GmbH 2020.

Supporting Information

Thienoisoindigo (TII)-Based Quinoidal Small Molecules for High-Performance n-Type Organic Field Effect Transistors

Arulmozhi Velusamy, Chih-Hsin Yu, Shakil N. Afraj, Chia-Chi Lin, Wei-Yu Lo, Chia-Jung Yeh, Ya-Wen Wu, Hsin-Chun Hsieh, Jianhua Chen, Gene-Hsiang Lee, Shih-Huang Tung, Cheng-Liang Liu, Ming-Chou Chen,* and Antonio Facchetti**

A. Velusamy, Dr. S. N. Afraj, W.-Y. Lo, C.-J. Yeh, Y.-W. Wu, H.-C. Hsieh, Prof. M.-C. Chen
Department of Chemistry and Research Center of New Generation Light Driven Photovoltaic Modules
National Central University
Taoyuan 32001, Taiwan
E-mail: mcchen@ncu.edu.tw

C.-H. Yu, C.-C. Lin
Department of Chemical and Materials Engineering
National Central University
Taoyuan, 32001 Taiwan

Prof. C.-L. Liu
Department of Materials Science and Engineering
National Taiwan University
Taipei 10617, Taiwan.
E-mail: liucl@ntu.edu.tw

Dr. G.-H. Lee
Instrumentation Center
National Taiwan University
Taipei 10617, Taiwan

Prof. S.-H. Tung
Institute of Polymer Science and Engineering
National Taiwan University
Taipei 10617, Taiwan

J. Chen, Prof. A. Facchetti
Department of Chemistry and the Materials Research Center
Northwestern University
Evanston, Illinois 60208, United States
Email: a-facchetti@northwestern.edu

Experimental Section

Materials

Starting materials (from Aldrich, Acros, or TCI Chemicals Co.) were reagent grade and were used without further purification unless otherwise indicated. Reaction solvents (toluene, ether, and tetrahydrofuran) were distilled under nitrogen from sodium/benzophenoneketyl, and halogenated solvents were distilled from CaH_2.

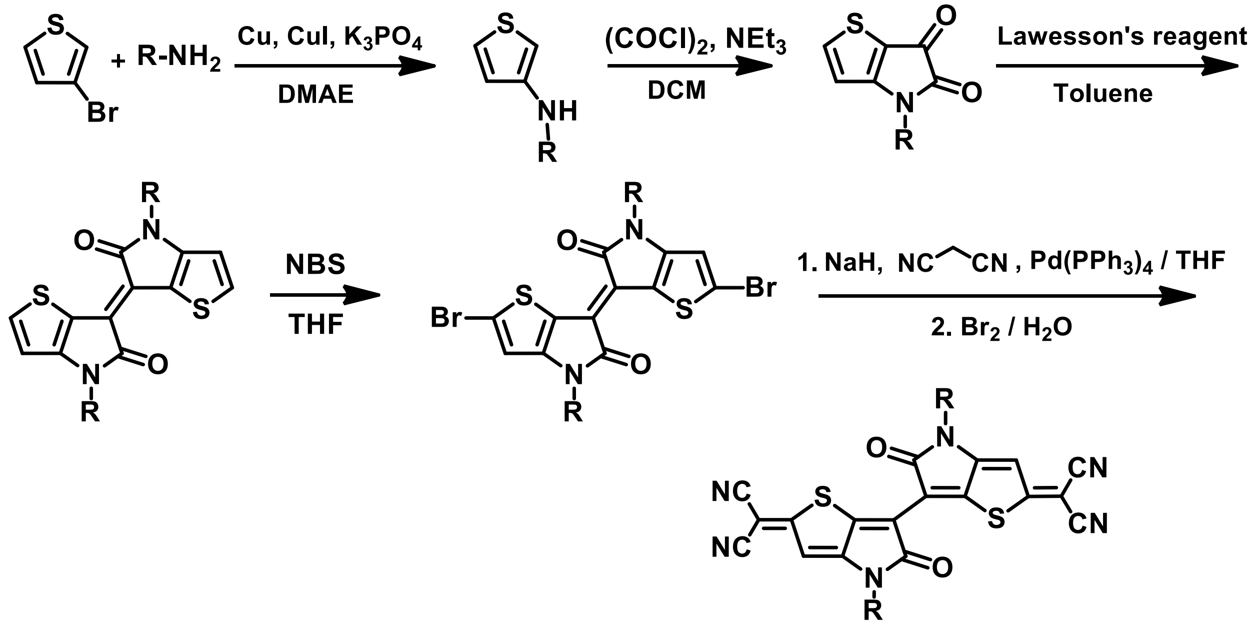

Scheme S1. Previous method to synthesize Quinoidal small molecules.[1-2]

General procedure for the synthesis of (6)

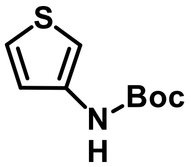

Under anhydrous and anaerobic conditions, 3-thiophenecarboxylic acid (0.50 g, 3.90 mmol), diphenyl phosphoryl chloride (1.36 g, 5.06 mmol) and NEt_3 (0.79 g, 7.81 mmol) in tert-butanol as a solvent were slowly added to the reaction flask at room temperature. After 1 hour, NaN_3 was added and heated at 80 °C for 15 hours. Then cooled to room temperature, the solvent was evaporated by a rotary concentrator and extracted with ethyl acetate. The organic layer was dried over $MgSO_4$ and then the solvent was evaporated and purified by column chromatography (eluent: ethyl acetate: n-hexane = 10:90).

The title compound was obtained as a White solid (yield = 50%). ^{1}H NMR (500 MHz, $CDCl_3$): δ 7.20 (d, *J* = 3.0 Hz, 1H), 7.19 (d, *J* = 3 Hz, 1H), 6.90 (d, *J* = 5.0 Hz, 1H), 6.66 (s, 1H), 1.51 (s, 9H).

General procedure for the synthesis of (7)

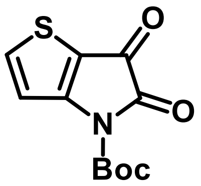

tert-butyl thiophen-3-ylcarbamate (0.24 g, 1.40 mmol) in DCM (10 mL) was added dropwise to oxalyl chloride (0.13 ml, 1.54 mmol) in DCM (20 mL) under anhydrous and anaerobic conditions at -15 °C. After reaction for 30 minutes, NEt_3 (0.26 g, 2.60 mmol) was added dropwise and the reaction was carried out at 0 °C for 1 hour. Further, the reaction was continued at room temperature for 12 hours. The reaction was quenched by the addition of water and extracted with dichloromethane. The organic layer was dried over $MgSO_4$ and then the solvent was evaporated.

The title compound was obtained as a Green solid (yield = 95%). ^{1}H NMR (500 MHz, $CDCl_3$): δ 8.05 (d, *J* = 5 Hz, 1H), 7.51 (d, *J* = 5 Hz, 1H), 1.63 (s, 9H).

General procedure for the synthesis of (8)

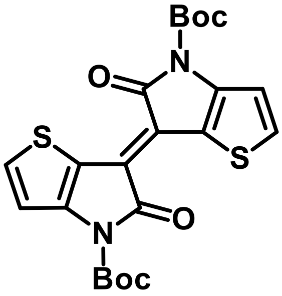

Under anhydrous and anaerobic conditions, *tert*-butyl 5,6-dioxo-5,6-dihydro-4*H*-thieno[3,2-*b*]pyrrole-4-carboxylate (TP-boc) (5.22 g, 20 mmol) reacts with Lawesson's reagent (4.18 g, 10 mmol) in 50 mL toluene at 60 °C for 12 hours. After completion of the reaction, the mixture was returned to room temperature, and the mixture was evaporated to dryness.

The title compound was obtained as a Dark red solid (yield = 50%). ^{1}H NMR (500 MHz, $CDCl_3$: δ 7.60 (d, *J* = 5 Hz, 2 H), 7.33 (d, *J* = 5 Hz, 2 H), 1.67 (s, 18 H).

General procedure for the synthesis of (9)

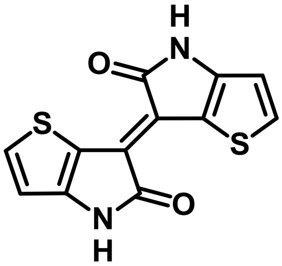

Trifluoroacetic acid (20 mL) was slowly dropped into (*E*)-di-*tert*-butyl 5,5'-dioxo-[6,6'-bithieno[3,2-*b*]pyrrolylidene]-4,4'(5*H*,5'*H*)-dicarboxylate (TII-boc) (0.20 g, 0.42 mmol) under anhydrous and anaerobic conditions at room temperature, reaction for 2 hours. Filter the solid and washed with deionized water.

The title compound was obtained as a Black solid (yield = 78%). ^{1}H NMR (500 MHz, d-DMSO): δ 10.91 (s, 1H), 7.78 (d, *J* = 5 Hz, 1H), 6.90 (d, *J* = 5 Hz, 1H).

General procedure for the synthesis of (10a-d)

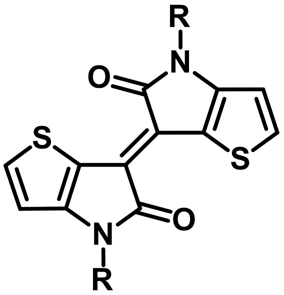

R = $C_{10}H_{21}$; 10a
bC_8H_{17} ; 10b
$bC_{16}H_{33}$; 10c
$bC_{17}H_{35}$; 10d

Under anhydrous and anaerobic conditions, (E)-[6,6'-bithieno[3,2-*b*]pyrrolylidene]-5,5'(4*H*,4'*H*)-dione (TII) (0.20 g, 0.73 mmol) in 20 ml DMF was added to K_2CO_3 (0.40 g, 2.9 mmol) in 10 ml DMF and reacted at 120 °C for 30 minutes. After the reaction was returned to room temperature, 1-bromo alkane (1.40 mmol) was added in 10 ml DMF and reacted at 120 °C for two hours. Then cooled to room temperature and concentrated under reduced pressure to remove solvent, extracted with ethyl acetate and the organic layer was dried over $MgSO_4$. Purification by chromatography (eluent: ethyl acetate: n-hexane = 20:80).

***Synthesis of 10a*:** The title compound was obtained as a Purple solid (yield = 52%). ^{1}H NMR (500 MHz, $CDCl_3$) : δ 7.53 (d, *J* = 5.5 Hz, 2 H), 6.81 (d, *J* = 5.5 Hz, 2 H), 3.80 (t, *J* = 7.5 Hz, 4 H), 1.73 (m, 4 H), 1.40~1.15 (m, 28 H), 0.86 (t, *J* = 7 Hz, 6 H).

***Synthesis of 10b*:** The title compound was obtained as a Purple solid (yield = 53%). ^{1}H NMR (500 MHz, $CDCl_3$): δ 7.55 (d, *J* = 5.5 Hz, 2 H), 6.82 (d, *J* = 5.5 Hz, 2 H), 3.73~3.70 (m, 4H), 1.88~1.86 (m, 2 H), 1.45~1.30 (m, 16 H), 0.95~0.88 (m, 12 H).

***Synthesis of 10c*:** The title compound was obtained as a Purple solid (yield = 55%). ^{1}H NMR (500 MHz, $CDCl_3$): δ 7.52 (d, *J* = 5.5 Hz, 2 H), 6.78 (d, *J* = 5.5 Hz, 2 H), 3.68 (d, *J* = 7 Hz, 4H), 1.88~1.86 (m, 2 H), 1.45~1.30 (m, 48 H), 0.87~0.83 (m, 12 H).

***Synthesis of 10d*:** The title compound was obtained as a Purple liquid (yield = 50%). ^{1}H NMR (500 MHz, $CDCl_3$): δ 7.52 (d, *J* = 5.5 Hz, 2 H), 6.79 (d, *J* = 5.5 Hz, 2 H), 3.81 (t, *J* = 7.5 Hz, 4 H), 1.65 (m, 4 H), 1.45~1.30 (m, 50 H), 0.89~0.86 (m, 12 H).

General procedure for the synthesis of (11a-d)

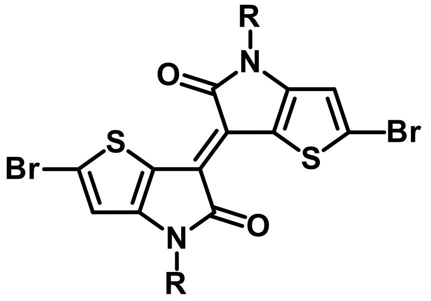

R = $C_{10}H_{21}$; 11a
bC_8H_{17} ; 11b
$bC_{16}H_{33}$; 11c
$bC_{17}H_{35}$; 11d

(*E*)-4,4'-dialkyl-[6,6'-bithieno[3,2-*b*]pyrrolylidene]-5,5'(4*H*,4'*H*)-dione (1.36 g, 1.81 mmol) was dissolved in 50 mL of THF under air, then NBS was slowly added (0.68 g, 3.81 mmol) at 0 °C, reaction for one hour at 0 °C, the color change from purple to blue-violet confirmed by TLC and the reaction was quenched with water and extracted with dichloromethane. The organic layer was dried over $MgSO_4$ and then the solvent was evaporated and recrystallized from ethanol.

Synthesis of 11a**:** The title compound was obtained as a Blue-Violet solid (yield = 85%). ^{1}H NMR (500 MHz, $CDCl_3$): δ 6.86 (s, 2 H), 3.75 (t, *J* = 7.5 Hz, 4 H), 1.69 (m, 4 H), 1.40~1.28 (m, 28 H), 0.87 (t, *J* = 7 Hz, 6 H)

Synthesis of 11b**:** The title compound was obtained as a Blue solid (yield = 85%). ^{1}H NMR (500 MHz, $CDCl_3$): δ 6.82 (s, 2 H), 3.74~3.62 (m, 4H), 1.78~1.77 (m, 2 H), 1.45~1.30 (m, 16 H), 0.95~0.88 (m, 12 H).

Synthesis of 11c**:** The title compound was obtained as a Blue solid (yield = 88%). ^{1}H NMR (500 MHz, $CDCl_3$): δ 6.81 (s, 2 H), 3.62 (d, *J*=7.5 Hz, 4H), 1.83 (m, 2 H), 1.28~1.23 (m, 48 H), 0.88~0.86 (m, 12 H).

Synthesis of 11d**:** The title compound was obtained as a Blue-Violet solid (yield = 85%). ^{1}H NMR (300 MHz, $CDCl_3$): δ 6.83 (s, 2 H), 3.77~3.72 (m, 4H), 1.78~1.50 (m, 4 H), 1.48~1.15 (m, 50 H), 0.89~0.85 (m, 12 H).

Table S1. Summary of Crystal Structure Data for **TIIQ-b16** (**3)**

Identification code	ic19459	
Empirical formula	C50 H68 N6 O2 S2	
Formula weight	849.22	
Temperature	200(2) K	
Wavelength	1.54178 Å	
Crystal system	Triclinic	
Space group	P-1	
Unit cell dimensions	a = 6.5305(2) Å	□= 100.3217(13)°.
	b = 10.4762(3) Å	□= 98.7190(13)°.
	c = 19.4150(5) Å	□ = 104.9005(11)°.
Volume	1235.24(6) $Å^3$	
Z	1	
Density (calculated)	1.142 Mg/m^3	
Absorption coefficient	1.305 mm^{-1}	
F(000)	458	
Crystal size	0.422 x 0.097 x 0.017 mm^3	
Theta range for data collection	4.486 to 74.972°.	
Index ranges	-8<=h<=7, -13<=k<=13, -24<=l<=24	
Reflections collected	9701	
Independent reflections	5070 [R(int) = 0.0237]	
Completeness to theta = 67.679°	99.8 %	
Absorption correction	Semi-empirical from equivalents	
Max. and min. transmission	0.7539 and 0.6315	
Refinement method	Full-matrix least-squares on F^2	
Data / restraints / parameters	5070 / 148 / 275	
Goodness-of-fit on F^2	1.096	
Final R indices [I>2sigma(I)]	R1 = 0.1088, wR2 = 0.3109	
R indices (all data)	R1 = 0.1205, wR2 = 0.3275	
Extinction coefficient	n/a	
Largest diff. peak and hole	0.712 and -0.641 e.$Å^{-3}$	

Crystallographic data (excluding structure factors) for the structure(s) reported in this paper have been deposited with the Cambridge Crystallographic Data Centre as supplementary publication no. **CCDC 1995257**

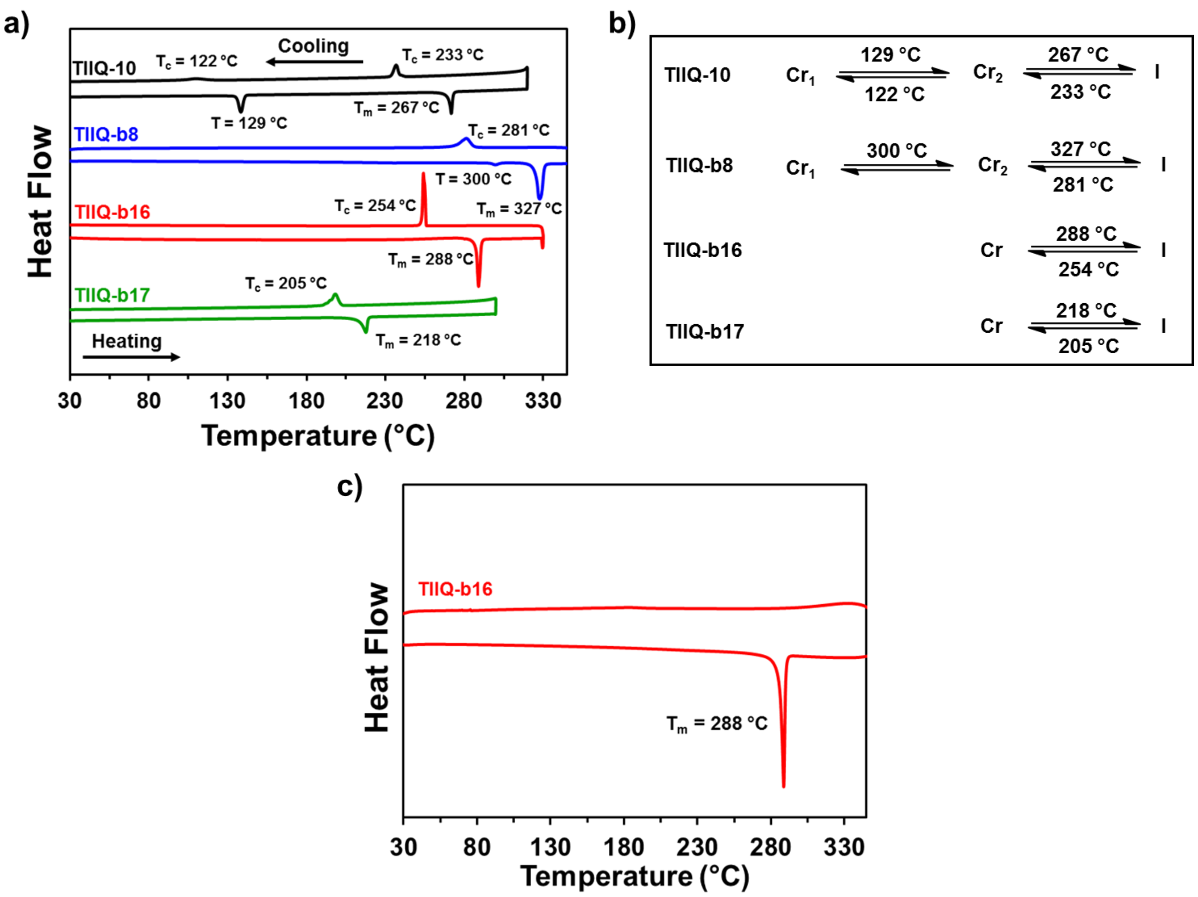

Figure S1. (a) DSC thermogram, (b) The phase transition temperatures of all TIIQs (Cr, Cr_1, Cr_2 = crystal phases and I = Isotropics, determined by DSC) and (c) Decomposition of TIIQ-b16 when heating upto 350 °C.

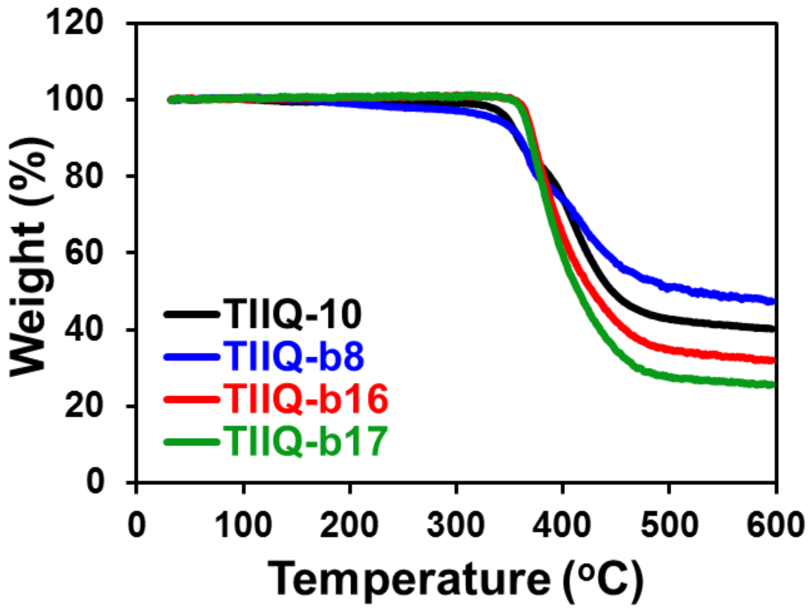

Figure S2. TGA thermogram of all TIIQs.

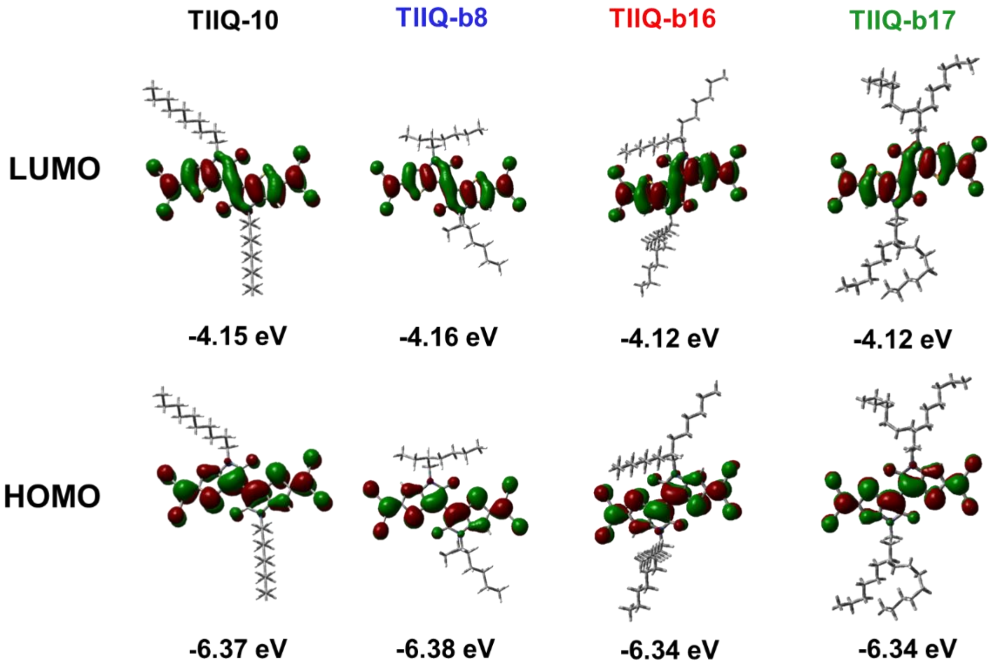

Figure S3. DFT-optimized geometries and patterns of HOMO and LUMO of TIIQs.

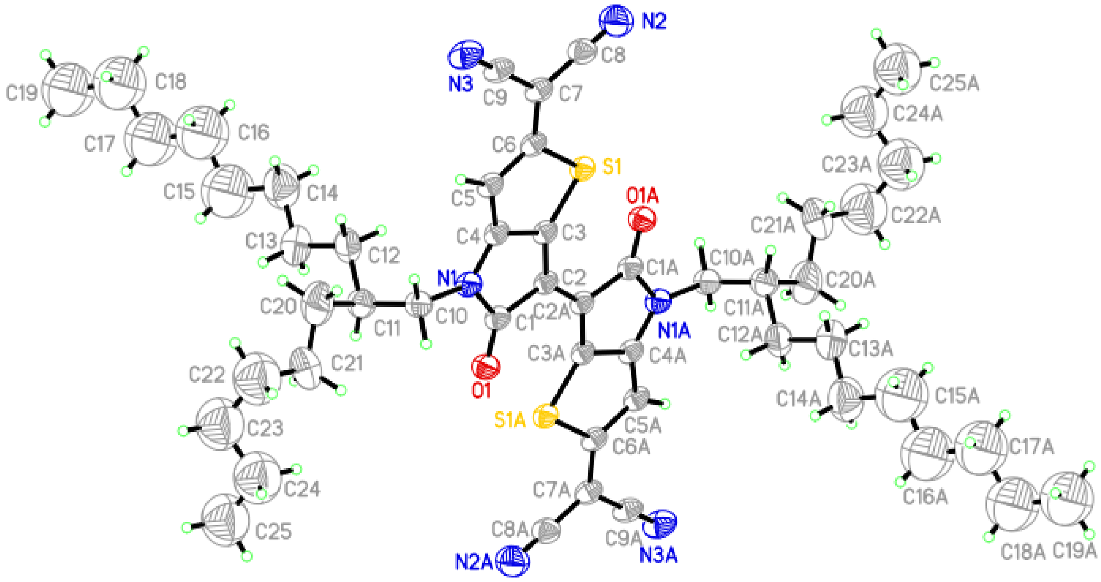

Figure S4. Single crystal structure of **TIIQ-b16**

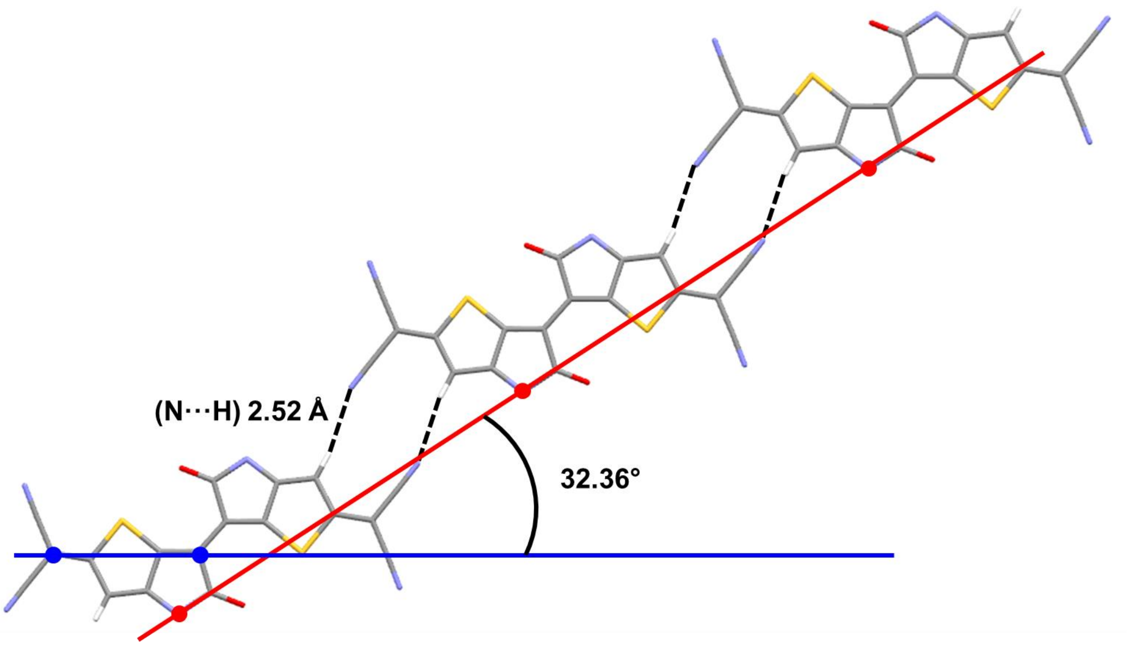

Figure S5. Intermolecular distance of **TIIQ-b16** measured between N···H 2.52 Å.

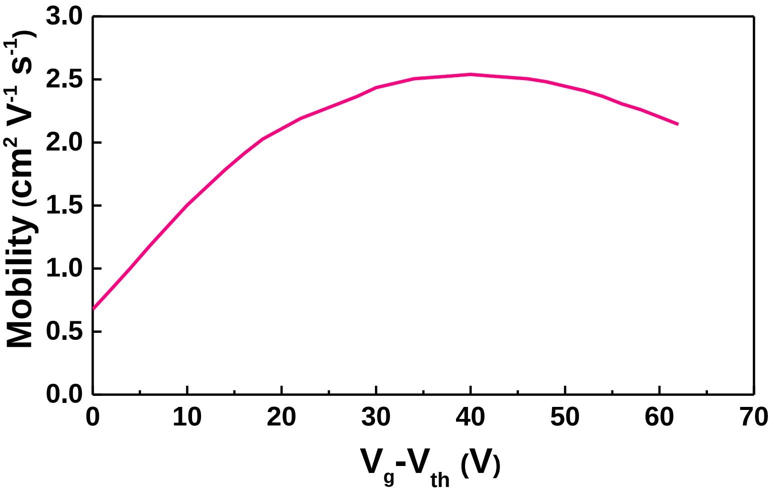

Figure S6. Mobility vs. (V_g-V_{th}) in saturation region.

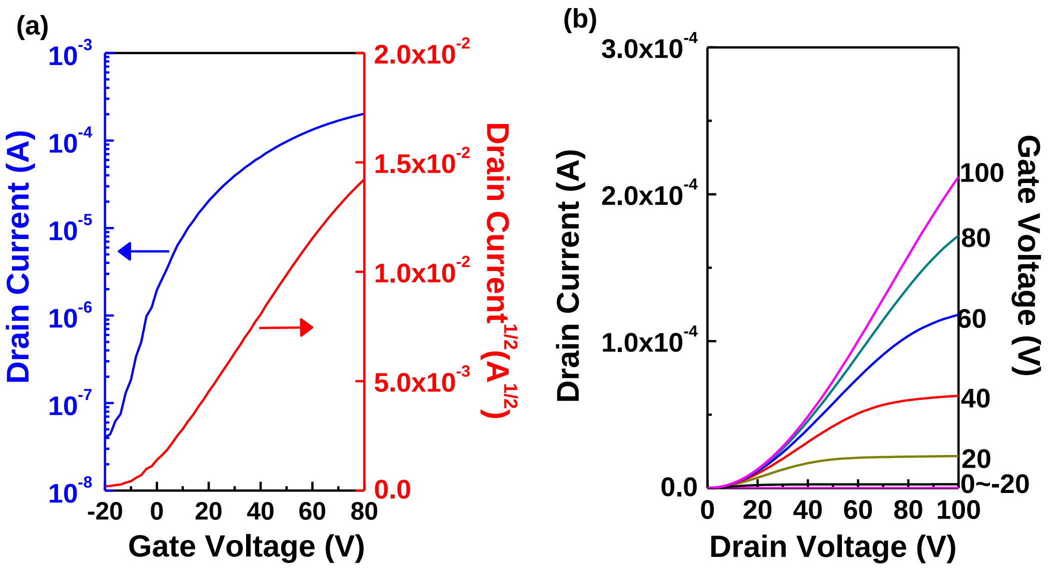

Figure S7. (a) Transfer and (b) output characteristics of **TIIQ-b16** OFETs fabricated by spin-coating.

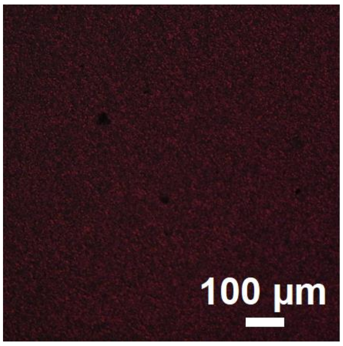

Figure S8. POM image of spin-coated **TIIQ-b16** film.

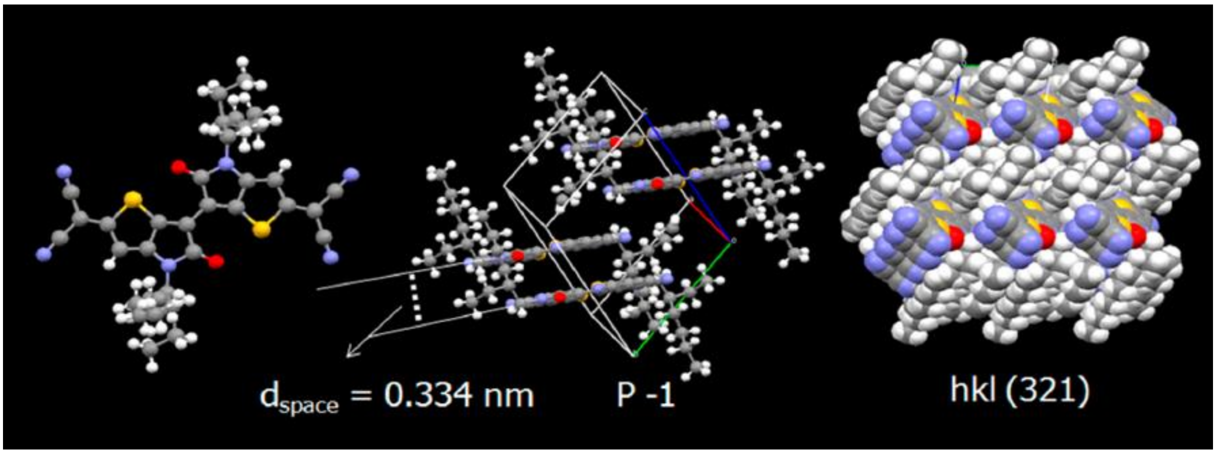

Figure S9. Single-crystal X-ray structure of DCN, which adopts a planar configuration and short π−π distance of 0.334 nm in a triclinic unit cell. A slice hkl(321), representing a lamella-like packing.[2]

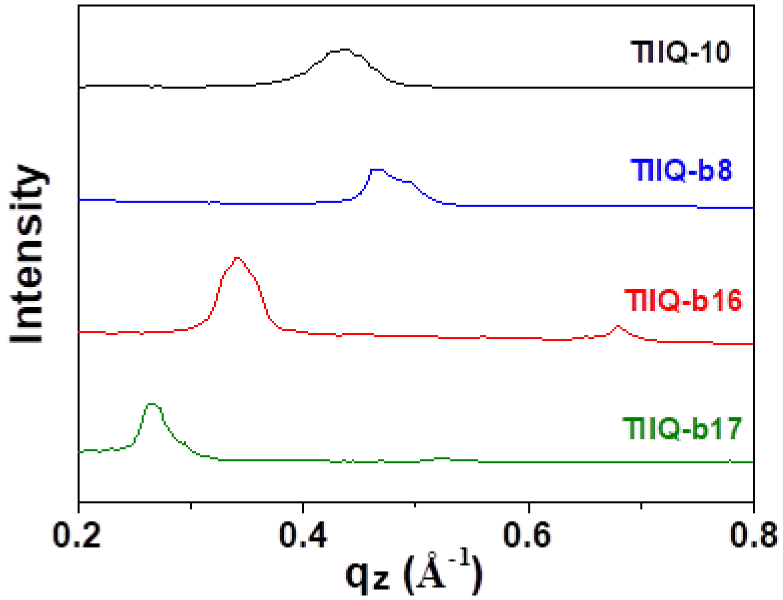

Figure S10. One-dimensional profile with respect to out-of-plane direction (along the q_z) direction extracted from GIXRD pattern of **Figure 9** in the main text.

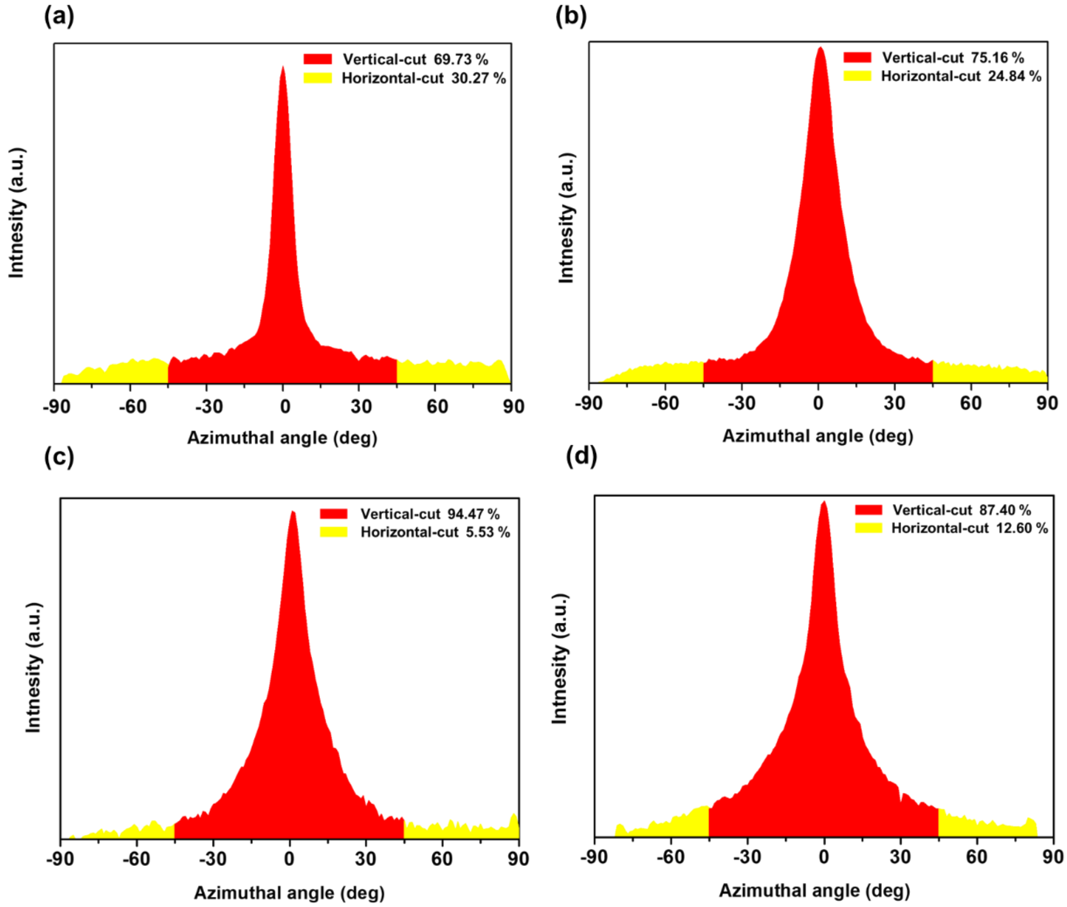

Figure S11. Pole figures for lamellar stacking (001) of (a) **TIIQ-10**, (b) **TIIQ-b8**, (c) **TIIQ-b16**, and (d) **TIIQ-b17** film.

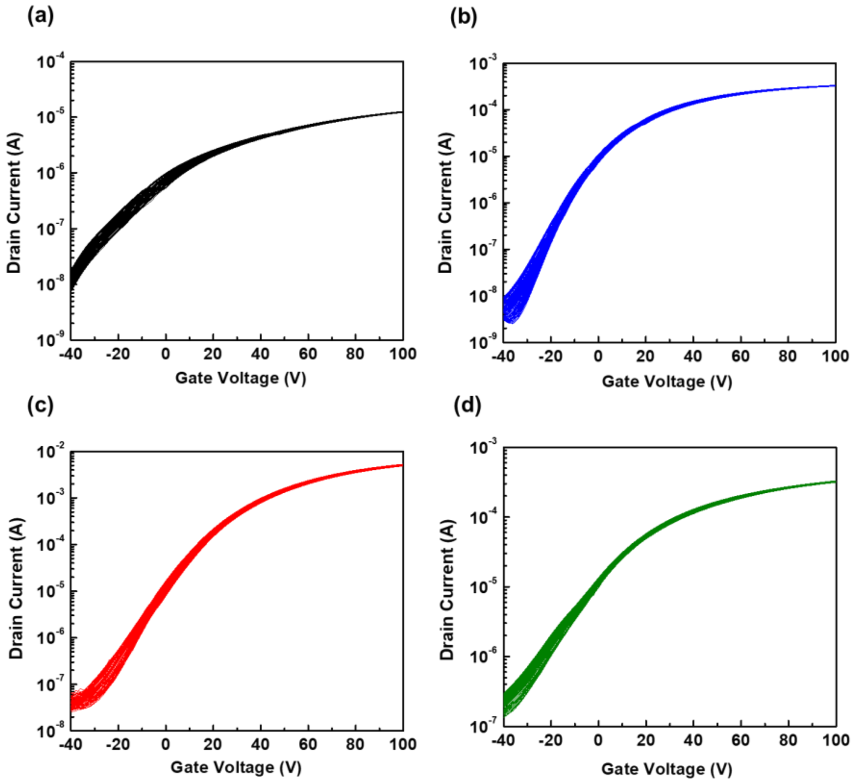

Figure S12. Continuous electrical transfer curve scan of solution-sheared (a) **TIIQ-10**, (b) **TIIQ-b8**, (c) **TIIQ-b16**, and (d) **TIIQ-b17** OFETs devices.

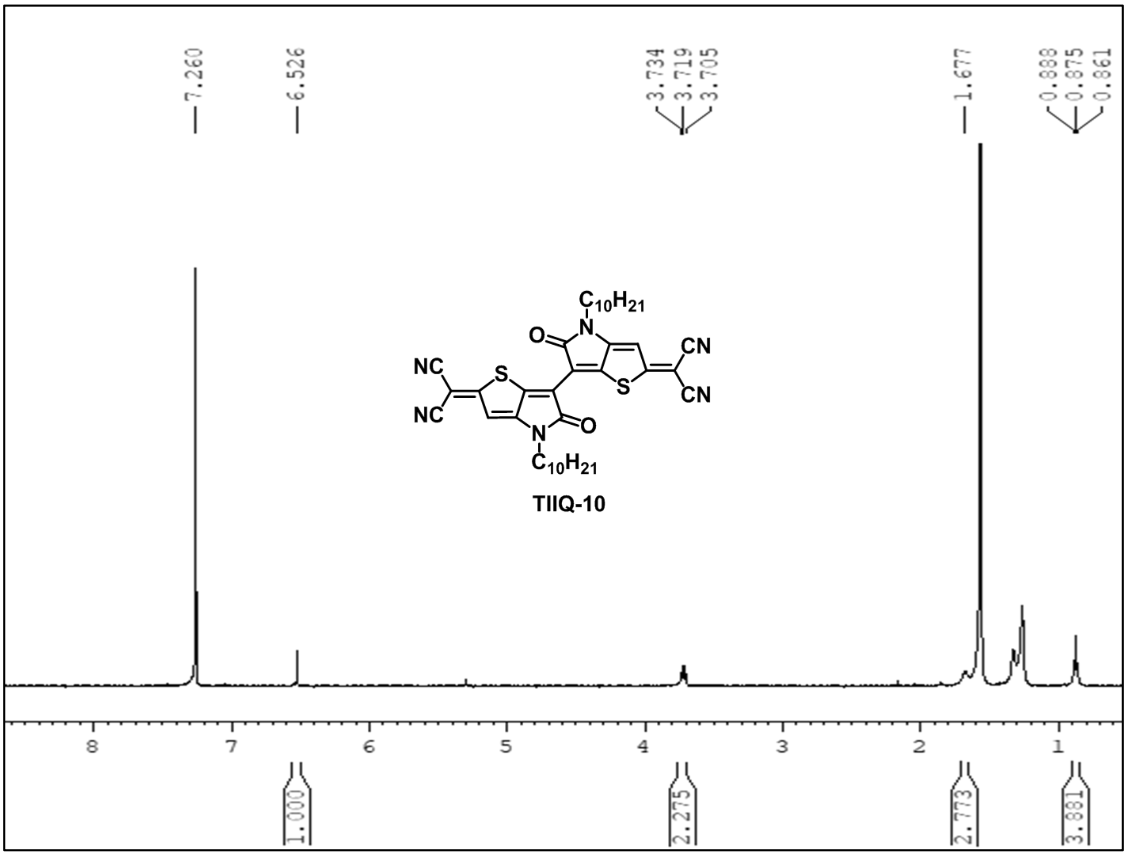

Figure S13. ^{1}H NMR spectrum of **TIIQ-10 (1)** in $CDCl_3$.

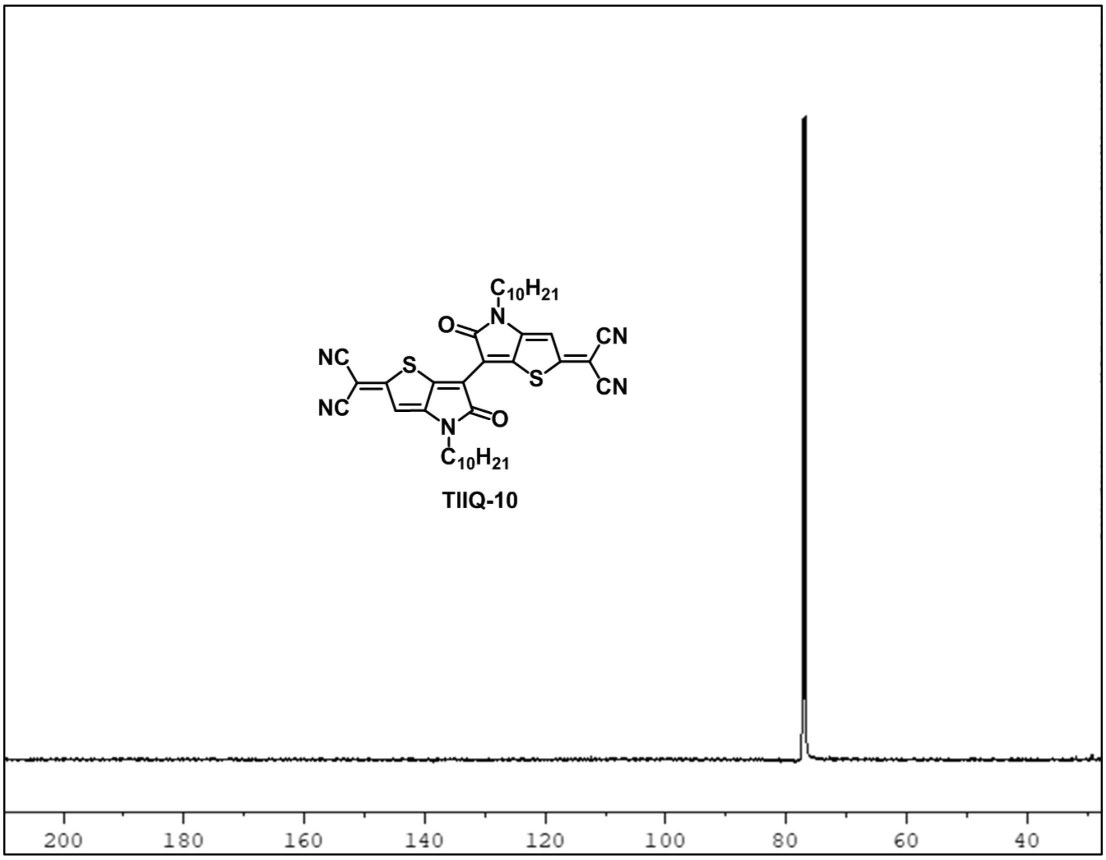

Figure S14. ^{13}C NMR spectrum of **TIIQ-10 (1)** in $CDCl_3$. (Insufficiently soluble)

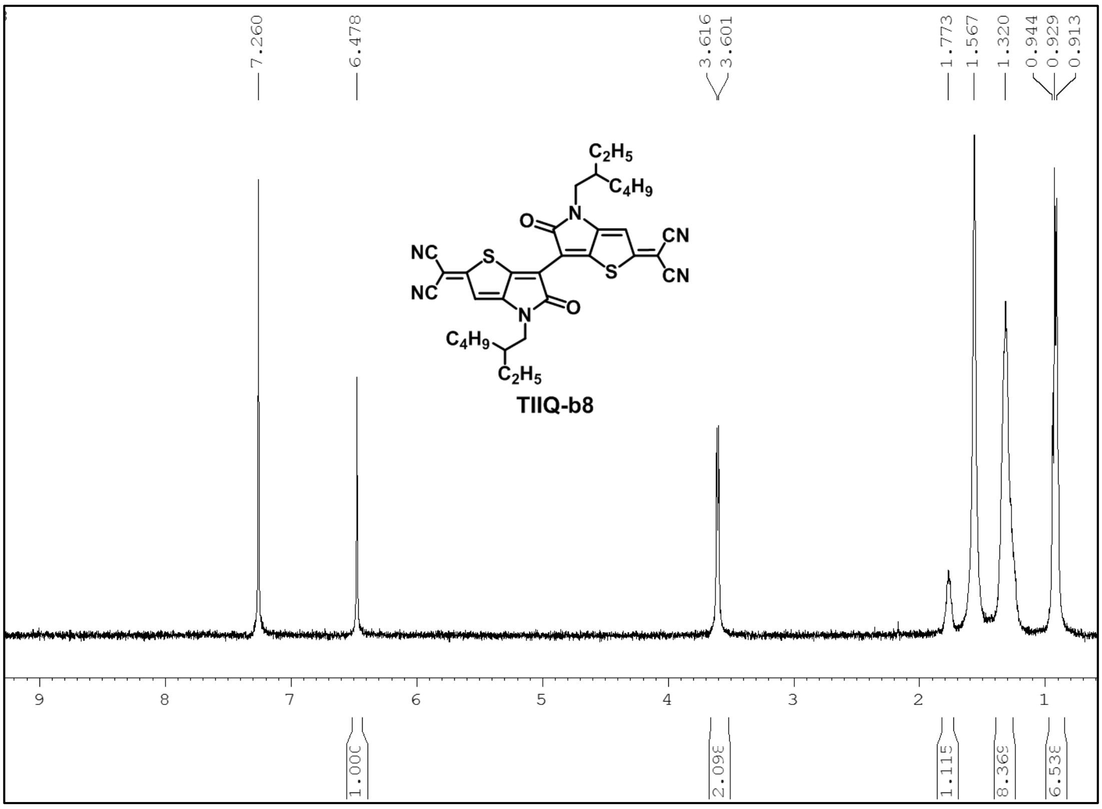

Figure S15. ^{1}H NMR spectrum of **TIIQ-b8 (2)** in $CDCl_3$.

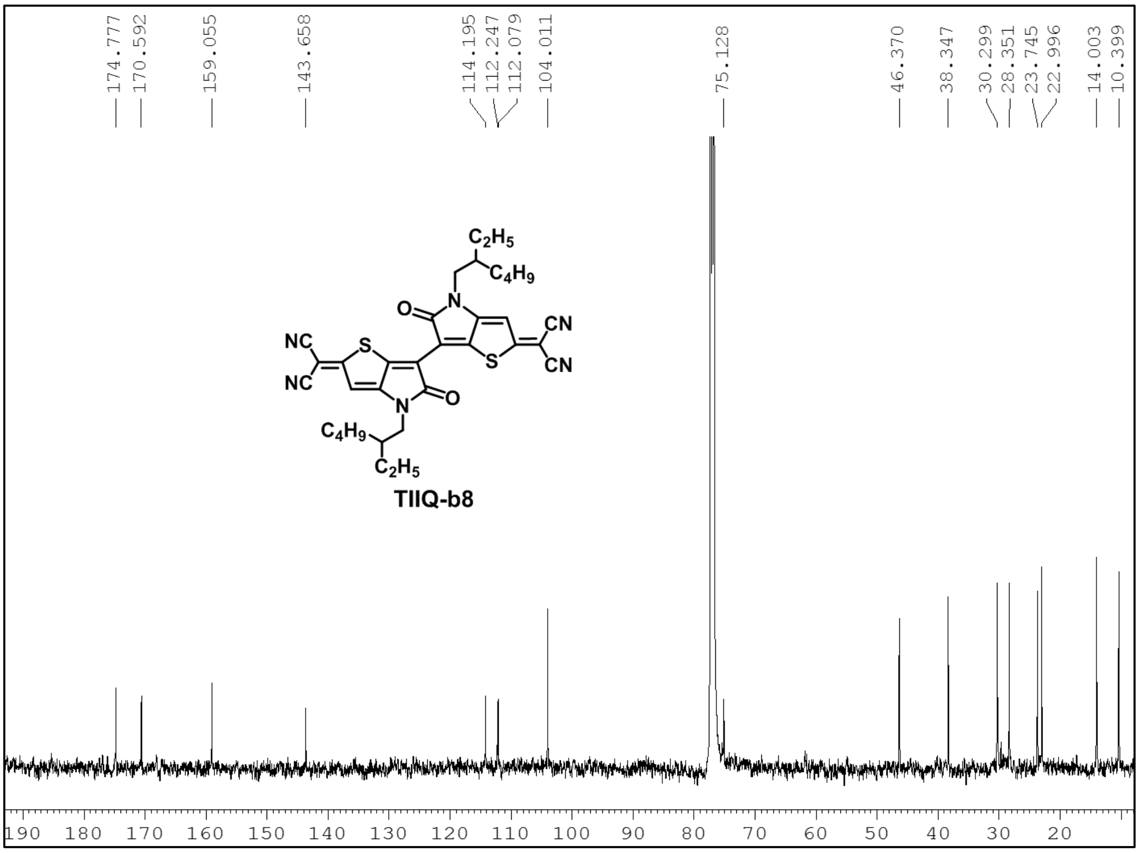

Figure S16. ^{13}C NMR spectrum of **TIIQ-b8 (2)** in $CDCl_3$.

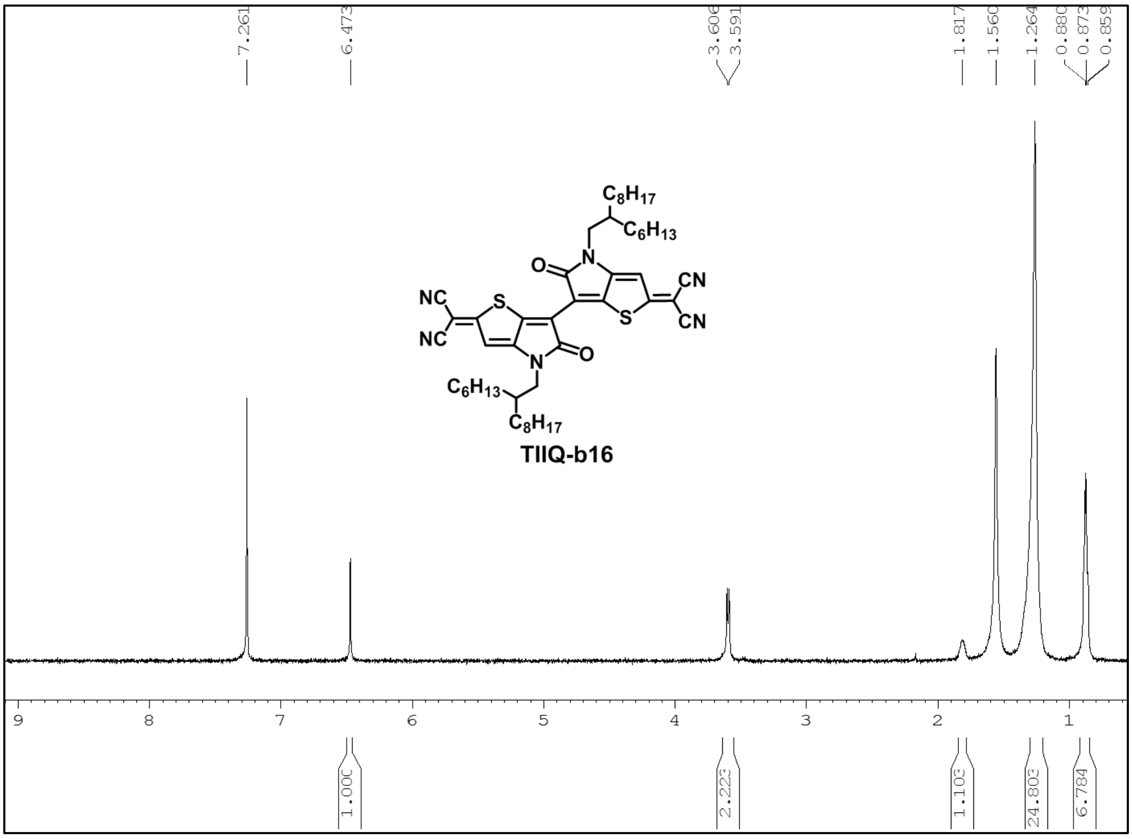

Figure S17. ^{1}H NMR spectrum of **TIIQ-b16 (3)** in $CDCl_3$.

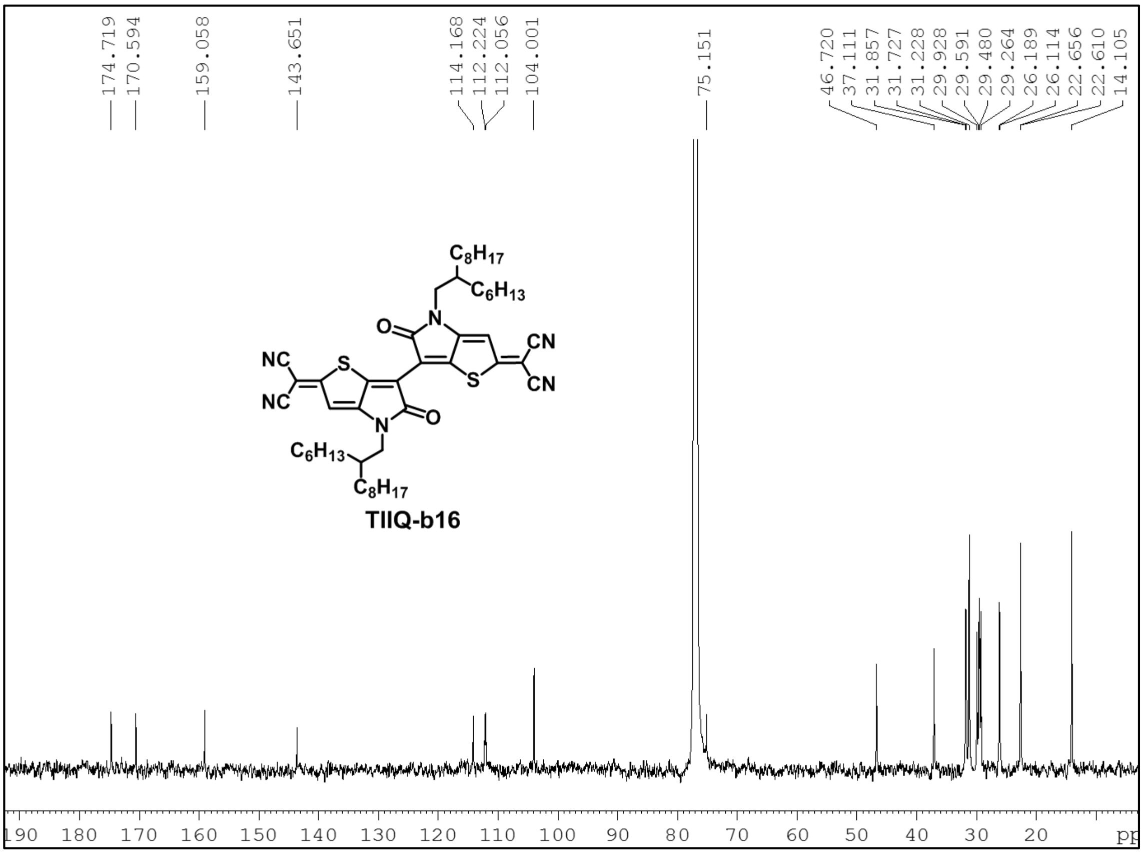

Figure S18. ^{13}C NMR spectrum of **TIIQ-b16 (3)** in $CDCl_3$.

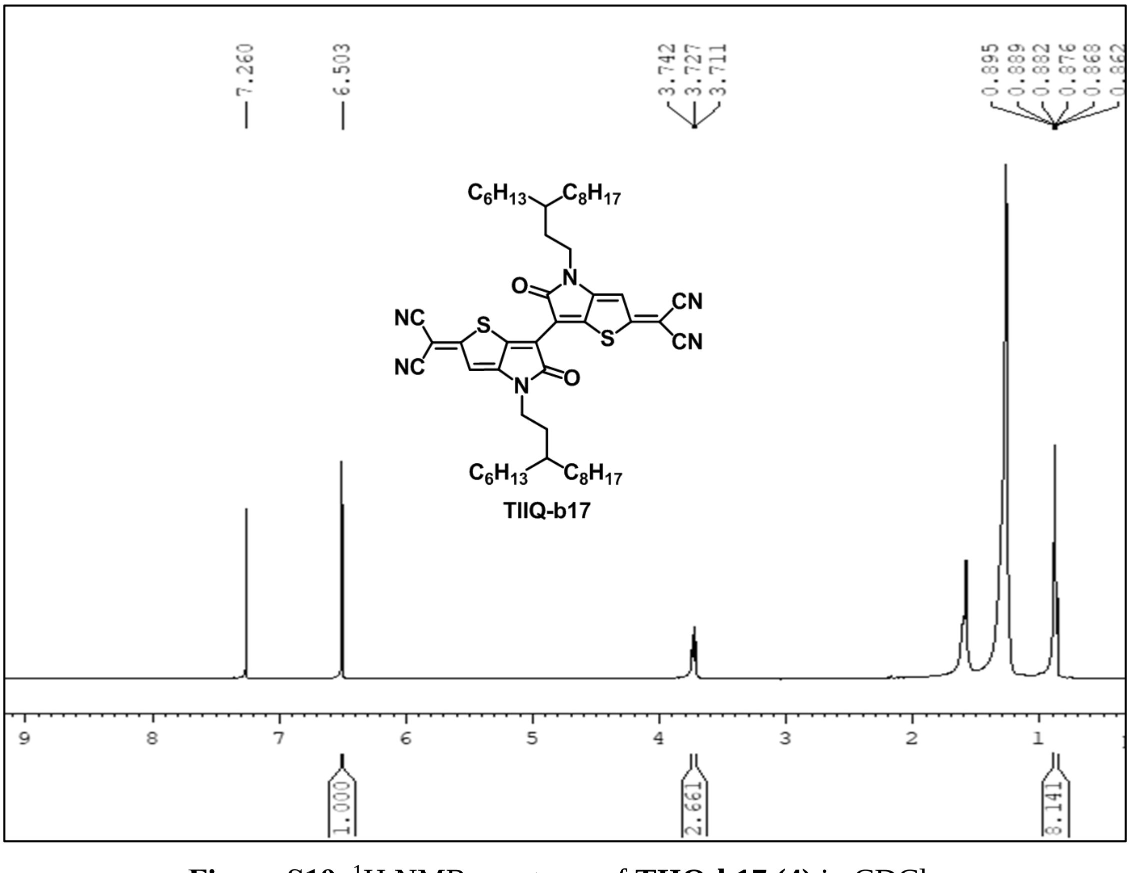

Figure S19. ^{1}H NMR spectrum of **TIIQ-b17 (4)** in $CDCl_3$.

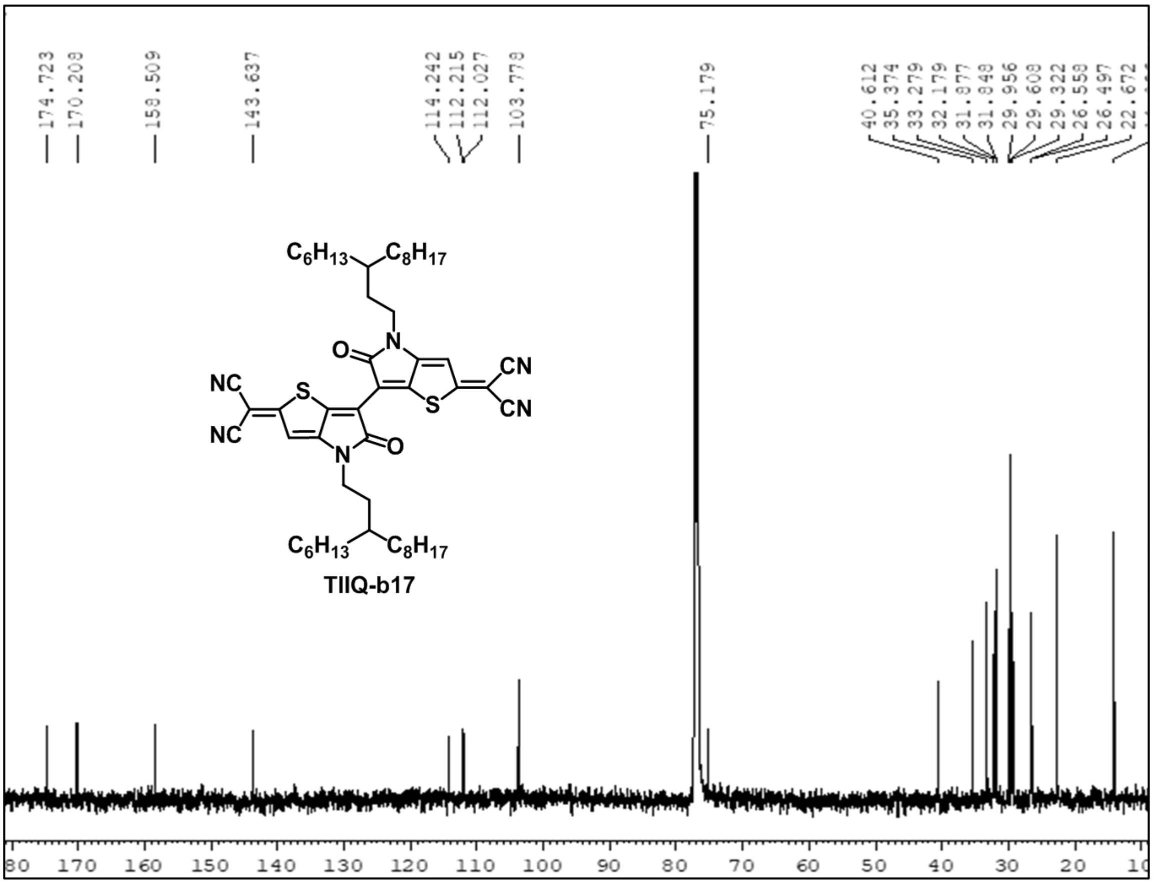

Figure S20. ^{13}C NMR spectrum of **TIIQ-b17 (4)** in $CDCl_3$.

References

[1] R. S. Ashraf, A. J. Kronemeijer, D. I. James, H. Sirringhaus, I. McCulloch, *Chem. Commun.* **2012**, *48*, 3939.

[2] C. C. Chueh, C. Z. Li, F. Ding, Z. Li, N. Cernetic, X. Li, A. K. Jen, *ACS Appl. Mater. Interfaces* **2017**, *9*, 1136.
